# Noncanonical MicroRNAs and Endogenous siRNAs in Lytic Infection of Murine Gammaherpesvirus

**DOI:** 10.1371/journal.pone.0047863

**Published:** 2012-10-26

**Authors:** Jing Xia, Weixiong Zhang

**Affiliations:** 1 Department of Computer Science and Engineering, Washington University, St. Louis, Missouri, United States of America; 2 Department of Genetics, Washington University School of Medicine, St. Louis, Missouri, United States of America; National Institute of Health, United States of America

## Abstract

MicroRNA (miRNA) and endogenous small interfering RNA (endo-siRNA) are two essential classes of small noncoding RNAs (sncRNAs) in eukaryotes. The class of miRNA is diverse and there exist noncanonical miRNAs that bypass the canonical miRNA biogenesis pathway. In order to identify noncanonical miRNAs and endo-siRNAs responding to virus infection and study their potential function, we sequenced small-RNA species from cells lytically infected with murine gammaherpesvirus 68 (MHV68). In addition to three novel canonical miRNAs in mouse, two antisense miRNAs in virus and 25 novel noncanonical miRNAs, including miRNAs derived from transfer RNAs, small nucleolar RNAs and introns, in the host were identified. These noncanonical miRNAs exhibited features distinct from that of canonical miRNAs in lengths of hairpins, base pairings and first nucleotide preference. Many of the novel miRNAs are conserved in mammals. Besides several known murine endo-siRNAs detected by the sequencing profiling, a novel locus in the mouse genome was identified to produce endo-siRNAs. This novel endo-siRNA locus is comprised of two tandem inverted B4 short interspersed nuclear elements (SINEs). Unexpectedly, the SINE-derived endo-siRNAs were found in a variety of sequencing data and virus-infected cells. Moreover, a murine miRNA was up-regulated more than 35 fold in infected than in mock-treated cells. The putative targets of the viral and the up-regulated murine miRNAs were potentially involved in processes of gene transcription and protein phosphorylation, and localized to membranes, suggesting their potential role in manipulating the host basal immune system during lytic infection. Our results extended the number of noncanonical miRNAs in mammals and shed new light on their potential functions of lytic infection of MHV68.

## Introduction

MicroRNAs (miRNAs) are ∼22-nt small noncoding RNAs (sncRNAs) that are encoded in virus, plants and animals [1]–[3]. They play essential regulatory roles in a wide variety of cellular processes. The canonical miRNA biogenesis consists of several steps involving RNase type III enzymes Drosha and Dicer [4]. Canonical miRNAs are initially transcribed by RNA Polymerase (RNA Pol) II as primary miRNA transcripts (pri-miRNAs) bearing foldback hairpin structures. The nuclear processing of pri-miRNAs by Drosha and double-strand RNA (dsRNA) binding protein Dgcr8 releases hairpin-structured precursors (pre-miRNAs). pre-miRNAs are recognized by Exportin5 and transported into cytoplasm. There, Dicer cleaves the pre-miRNAs to release ∼22-nt RNA duplexes with ∼2-nt 3′ overhangs. One strand of a RNA duplex, termed mature miRNA, is subsequently loaded into the Argonaute-containing (AGO) RNA-induced silencing complex (RISC). The miRNA guides the RISC to its perfectly or partially complementary binding sites, which are normally in the 3′ untranslated regions (UTRs) of targeted transcripts in animal organisms, to exert its regulatory function. The binding preference often depends on the miRNA's 2- to 8-nt sequence from its 5′ end, the so-called seed region of the miRNA [5].

miRNA biogenesis is complex; a collection of diverse miRNAs can be generated through noncanonical pathways that bypass Drosha/Dgcr8 processing. One example is the class of miRtrons [6], [7], which are derived from debranched intron lariats serving as pre-miRNAs, for which Spliceosome functions as Drosha to release pre-miRNAs. miRtrons can be further categorized into typical miRtrons, which are derived from the whole regions of short introns, and tailed miRtrons, which are generated from end regions of longer introns [8], [9]. Previous studies of mammalian miRtrons identify three typical miRtrons, which are well conserved in mammals [10]. The most prominent example is miR-877, a typical miRtron whose miRNA and miRNA* sequences reside near the exon/intron boundaries.

Another type of noncanonical miRNAs originates from small nucleolar RNAs (snoRNAs) through their internal hairpin-shaped folding structures [11]. Processing of snoRNA-derived hairpins gives rise to Dicer dependent but Drosha-/Dgcr8-independent miRNAs. Besides snoRNAs, transfer RNA (tRNA) transcripts may also fold into alternative hairpin structures, on which Dicer exerts cleavage activities without otherwise precedent processing of Drosha/Dgcr8 [12], [13]. Another origin of noncanonical miRNAs arises from endogenous short hairpin RNAs (shRNAs), from which transcripts originating from unannotated, intergenic regions can serve as Dicer substrates upon transcription [8], [14]. In certain cases, miRNA biogenesis depends on Drosha but bypasses Dicer processing where AGO-containing RISC substitutes Dicer for processing pre-miRNAs [15].

Over 200 viral miRNAs have been reported for a variety of DNA viruses, predominately in the Herpesviridae family. These include 25 miRNAs in human Epstein-Barr virus (EBV) and 12 in Kaposi's sarcoma herpesvirus (KSHV) [16]. Similar to EBV and KSHV, murine gammaherpesvirus 68 (MHV68) encodes 15 distinct miRNAs, tightly located within the first 10 kb region of the MHV68 genome [17]–[19]. However, different from EBV and KSHV, MHV68 generates these primary miRNA transcripts via RNA Pol III, instead of RNA Pol II, as part of larger transcripts that include tRNA-like units directly upstream of the miRNAs [17]–[19]. Therefore, these miRNAs are generated via a noncanonical miRNA pathway that utilizes RNase Z instead of Drosha to liberate pre-miRNAs from tRNAs [20].

In contrast to miRNAs, endo-siRNAs are derived from long dsRNAs in the form of annealed natural antisense transcripts (NATs) or long hairpin RNAs (hp-RNAs). Processing of long dsRNAs is directly performed by Dicer along dsRNAs to consecutively produce multiple siRNAs. Diverse sources for dsRNA formation have been identified in animals and plants; for review, see ref [21]. One source of dsRNAs arises from tandem inverted repeats. For example, folding of a transcript of two tandem inverted B1 SINEs may yield a long hairpin substrate, which can be subjected to Dicer cleavage without Drosha/Dgcr8 activities [12]. Multiple species of 21- to 22-nt endo-siRNAs can be generated via a sequential cleavage activities of Dicer along a long hairpin, producing siRNAs of 21- to 22-nt arranged in phase [12]. While miRNAs are widely expressed in various cells and tissues, currently endo-siRNAs have only been reported to originate from mouse stem cells [12], [22] and oocytes [23], [24].

In this study, we prepared and sequenced small-RNA libraries of cells lytically infected with MHV68 and of mock-treated cells, and searched for canonical and noncanonical miRNAs as well as endo-siRNAs in the MHV68 and mouse genomes. A total of 30 novel miRNAs were identified, which include two antisense miRNAs (miRNAs on the antisense strands of known miRNAs) in the MHV68 genome, and 3 canonical miRNAs and 25 noncanonical miRNAs derived from tRNAs, snoRNAs and introns of the mouse genome. The hairpin structures of virus pre-miRNAs and that of snoRNA- and shRNA-derived pre-miRNAs in mouse were observed to lack loop-distal regions and to be shorter than canonical miRNA hairpins. Atypical base pairings outside the seed regions and a predominant preference of Adenosine as the first nucleotides were observed for noncanonical miRNAs. Furthermore, we also identified a novel endo-siRNA-generating locus in a B4 SINE-inverted repeat region of the mouse genome. Moreover, a set of endo-siRNAs was found in a collection of our data from MHV68-infected cells and previously published sequencing data. Besides the expression of all known and novel miRNAs in the virus, a noncanonical miRNA in mouse was significantly up-regulated in infected cells than in mock-treated cells. The viral and highly up-related miRNAs were predicted to have a large number of target genes, many of which were involved in processes of gene transcription and protein phosphorylation and localized to cell membranes, suggesting their potential role in manipulating the host basal immune system during lytic infection.

## Results

### Small-RNA profiling by deep sequencing

Three MHV68-infected and two mock-treated small-RNA libraries were prepared from NIH 3T12 cells. The five small-RNA libraries were sequenced separately using Illumina Genome Analyzer II. Both the biological triplet of infected samples and the biological duplicate of mock samples were of a high fidelity and the deep sequencing experiments were highly reproducible; the Pearson's correlation coefficients of a pair of biological duplicates range from 0.8939 to 0.9849. The deep-sequencing profiling experiments resulted in a total of more than 74 million raw sequence reads and 28,268,434 qualified sequence reads, among which 274,266 mapped to the MHV68 genome and 21,364,827 aligned to the mouse reference genome and cDNAs perfectly with no mismatches. Of the reads mapped to MHV68, 241,683 (88.1% of the total) came from the known MHV68 pre-miRNA sequences; of the reads mapped to the host genome, 19,701,917 (92.2% of the total) were from the known murine pre-miRNA sequences. In total, the currently annotated MHV68 and host miRNAs produced 92% of the total mappable reads present in 3T12 cells (Tables S1 and S2). The remaining reads either represented fragments of cellular mRNAs or noncoding RNAs (rRNAs, tRNAs, snoRNAs, etc.) or were from currently annotated repeats regions (see Methods). Finally, we also detected 32,583 reads that mapped to the MHV68 genome but not to any of the 15 known MHV68 pre-miRNAs. All of these reads mapped to tRNA fragments or annotated open reading frames (ORFs) of MHV68.

### miRNAs discovery in the virus and mouse genomes

We performed a comprehensive search of new miRNA genes in the virus and mouse genomes (see Methods) based on the following criteria: (1) occurrence of at least ten normalized reads on the arm of a predicted hairpin structure; (2) presence of a miRNA/miRNA* duplex with 1- to 3-nt 3′ overhang if miRNA* sequence appear; (3) non-uniform distribution of reads length with peak of 20- to 24-nt; (4) Dicer dependency by examining the data of dicer-knockout mice, particularly when criterion (2) above was not met; and (5) presence of reads aligned to an intron/exon boundary for identifying a miRtron. In some cases, miRNA* sequences might not be detected because of their low abundance. In a previous study, for example, conserved miR-184 and miR-489 were initially ruled out due to the lack of miRNA* reads in sequencing data but were subsequently rescued by experimental validation [8]. Therefore, instead of absolutely obeying the miRNA* rule, we further examined the property of Dicer dependency for each of the new miRNA candidates by analyzing two publicly available data sets of wild-type and dicer1-knockout mice [12]. Note that our miRNA criteria followed in principle the criteria set forth previously [25]. A flowchart describing the procedure of miRNAs identification is provided in Figure S1.

A total of 30 novel miRNAs were identified in the virus and host genomes, including two virus miRNAs and 28 murine miRNAs (Table 1 and File S1). The new murine miRNAs can be further divided into three canonical and 25 noncanonical miRNAs, including nine snoRNA-derived and one tRNA-derived miRNAs as well as 15 miRtrons. Combined with 2 known miRNAs newly annotated as miRtrons in this study and 30 previously annotated noncanonical miRNAs [8], [12], at least 57 murine noncanonical miRNAs exist (Tables 1, S3 and S4).

**Table 1:** Thirty novel miRNAs in the MHV68 or mouse genomes.

novel miRNA ID	type	# Reads^a^	Rank^b^	location	chrom	start	end	strand
mghv-miR-M1-8-AS	virus	12	15*	miR-M1-8 (antisense)	MHV68	3813	3871	−
mghv-miR-M1-10-AS	virus	10	16*	miR-M1-10 (antisense)	MHV68	260	321	−
Murine miRNAs								
novel-miR-#1	canonical	247	227	HFIC (intron)	chr10	80878974	80879053	−
novel-miR-#2	canonical	34	364	Ncor2 (intron)	chr5	125574701	125574798	−
novel-miR-#3	canonical	343	197	Tmem123 (intron)	chr9	7784378	7784454	+
sno-miR-#1	snoRNA-derived	549	179	SNORA18 (H/AC A)	chr9	15118311	15118375	+
sno-miR-#2	snoRNA-derived	23	388	SNORA76 (H/ACA)	chr11	106362626	106362688	+
sno-miR-#3	snoRNA-derived	1159	149	SNORA7A (H/ACA)	chr6	115757994	115758062	−
sno-miR-#4	snoRNA-derived	150	267	SNORA41 (H/ACA)	chr1	63225644	63225708	+
sno-miR-#5	snoRNA-derived	80	312	SNORA5 (H/ACA)	chr11	6519815	6519875	−
sno-miR-#6	snoRNA-derived	46	340	SNORA25 (H/ACA)	chr13	62238357	62238421	+
sno-miR-#7	snoRNA-derived	577	177	SNORA44 (H/ACA)	chr4	131865918	131865980	+
sno-miR-#8	snoRNA-derived	43	345	SNORA2 (H/ACA)	chr15	37988103	37988169	+
sno-miR-#9	snoRNA-derived	36	356	SNORA1 (H/ACA)	chr9	15119701	15119764	+
tRNA-miR-#1	tRNA-derived	12	433	tRNA-SerAGA	chr13	23592811	23592912	−
miRtron-#1	tailed miRtron	11	440	Snrnp70	chr7	52639256	52639324	−
miRtron-#2	tailed miRtron	10	446	Kifc1	chr17	27057908	27057964	+
miRtron-#3	tailed miRtron	135	284	Troap	chr15	98911238	98911296	+
miRtron-#4	tailed miRtron	51	333	Cnot1	chr8	98277051	98277114	−
miRtron-#5	tailed miRtron	10	447	Ptprf	chr4	117883110	117883169	−
miRtron-#6	miRtron	10	448	Man2c1	chr9	56989723	56989801	+
miRtron-#7	tailed miRtron	18	406	Supt5h	chr7	29114504	29114569	−
miRtron-#8	tailed miRtron	10	449	Wiz	chr17	32499909	32499969	−
miRtron-#9	tailed miRtron	19	404	Pom121	chr5	135859599	135859677	−
miRtron-#10	tailed miRtron	10	450	Supt6h	chr11	78039600	78039669	−
miRtron-#11	tailed miRtron	10	451	Dohh	chr10	80849094	80849157	+
miRtron-#12	tailed miRtron	11	441	Dazap1	chr10	79740909	79740977	+
miRtron-#13	miRtron	15	420	Skiv2l	chr17	34982046	34982112	−
miRtron-#14	miRtron	13	430	Plcb3	chr19	7034708	7034781	−
miRtron-#15	tailed miRtron	11	442	Dock6	chr9	21614384	21614443	−

a The number of reads perfectly mapping to pre-miRNA hairpin sequences in all samples.

b The rank of miRNAs relative to all known and novel murine miRNAs.

* The rank of miRNAs relative to all the MHV68 miRNAs.

### Antisense miRNAs and isomiRs in the MHV68 genome

**Figure 1:**
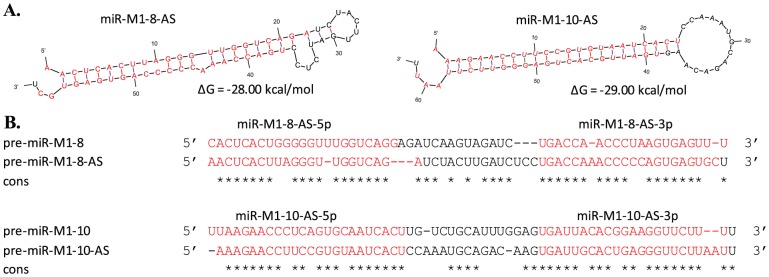
Two novel MHV68 antisense miRNAs. (A) Hairpin structures of miR-M1-8-AS and miR-M1-10-AS with the miRNAs annotated in red on both arms. (B) Sequence similarities between sense and antisense precursors of miR-M1-8-AS and miR-M1-10-AS where the miRNA sequences are annotated in red. The color bar on the last line shows the similarity between this pair of sense and antisense miRNAs.

Previously, we used part of the sequencing data that is presented here to identify five novel miRNAs (miR-M1-10, -12 to -15) in the MHV68 genome [18]. Using a larger sequencing dataset in the current study, we were able to identify two novel antisense miRNAs in the virus genome, namely miR-M1-8-AS and miR-M1-10-AS (Figure 1A and Table 1), which reside antisense to miR-M1-8 and miR-M1-10, respectively. Three pieces of evidence supported that these two new miRNAs are authentic. First, folding of the sequences surrounding the two antisense miRNAs yielded hairpin structures, which are the structural signature of miRNAs. Second, alignment of sequence reads to the hairpin structures revealed RNA-RNA duplexes with ∼2-nt 3′ overhangs (Figure 1A), which are indicative of Dicer cleavage activities. Third, no sequence reads mapped to the loop regions of the hairpins, suggesting that the antisense miRNAs originated from the hairpins rather than from possible dsRNAs formed by annealing of the sense and antisense transcripts.

Both miR-M1-8-AS and miR-M1-10-AS were distinct from their sense counterparts with regard to precursor and mature miRNA sequences. Nevertheless, the sense and antisense miRNA precursors also shared high sequence similarities (Figure 1B). Both miR-M1-8-AS and miR-M1-10-AS have fewer sequence reads than their sense miRNAs, suggesting that the antisense miRNAs might result from coincidental transcription.

miRNA sequence variances, known as miRNA isoforms or isomiRs, have been observed in previous studies [18], [26], [27]. miRNA isoforms can have important implications for their target gene regulation since their seed regions are shifted [8]. Unlike conserved murine miRNAs, we observed that one third of MHV68 encoded miRNAs yielded 5′ isoforms that shifted from the annotated MHV68 miRNAs (miRbase version 17) by 1- to 3-nt. The most prominent example is miR-M1-2-5p, where two species of 5′ isoforms shift from the annotated mature miR-M1-2-5p by 1- and 2-nt, respectively. In total, miR-M1-2-5p and its two 5′ isoforms, which produced more than 5,000 reads each, comprised up to 40%, 34% and 23% of the reads mapped to the miR-M1-2 pre-miRNA, respectively (Table S2).

### Diverse miRNAs in the mouse genome

#### Canonical miRNAs

We identified 3 novel canonical miRNAs in the mouse genome (Table 1). Despite that they were expressed at low abundance, one of them (novel-miR-#1) had miRNA* reads and formed a miRNA/miRNA* duplex with 2-nt 3′ overhang. Although the other two novel miRNAs did not have miRNA* reads, the sequences of these three novel miRNAs appeared in the sequencing data of wild-type mice but not in the data of dicer1- or dgcr8-knockouts [12], [13], indicating that they are Dicer dependent and thus are genuine miRNAs (Table S3). As for genomic distribution, all of the three novel miRNAs were located in introns of their host genes (Table 1). Furthermore, we identified a set of miRNA candidates, listed in Table S5 and File S1. Classification of their biogenesis as canonical or noncanonical miRNAs needs further investigation due to their relatively low sequencing abundance to determine their enzyme dependency.

#### Noncanonical miRNAs derived from snoRNAs

**Figure 2:**
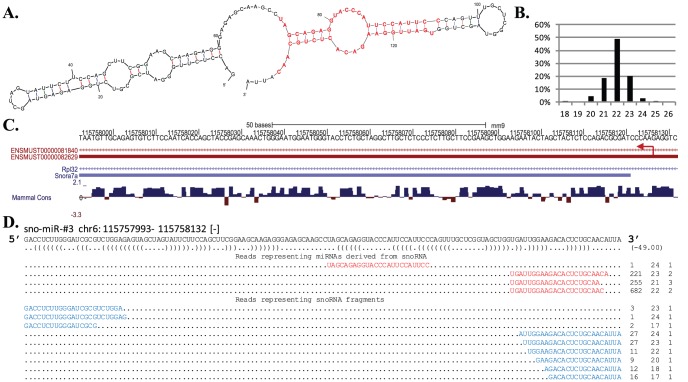
A murine miRNA, sno-miR-#3, derived from snoRNA Snora7a. (A) Folding structure of the snoRNA carrying a hairpin shaped structure. The miRNAs (annotated in red) on 5′ and 3′ arm form a duplex with 1-nt 3′ overhang. (B) Length distribution of the total reads mapped to the snoRNA, which peaked at 22-nt. (C) snoRNA gene annotated in Ensembl and RefSeq annotations. Snora7a resides within an intron of Rpl32. The red arrow indicates the orientation of the snoRNA gene. The conservation score by PhyloP shows how well each base pair is conserved in mammals. (D) Alignment of reads representing miRNA sequences (red) and snoRNA fragments (blue), which appeared in Dicer- and Dgrc8-knockout mice.

We recognized nine H/ACA snoRNA loci in the mouse genome harboring novel noncanonical miRNAs (Table 1). The miRNAs from these loci are genuine noncanonical miRNAs due to their hairpin structures (Figure 2A; File S1), length distributions which peak at 22-nt (Figure 2B), and the property of Dicer-dependency but, critically, Dgcr8-independency (Figure S2 and Table S3). A prominent example is sno-miR-#3, which is located within snoRNA *Snora7a* and yielded 1,159 sequencing reads (Figure 2C). Moreover, six of the nine snoRNA-derived miRNAs appeared to be well conserved in mammals, indicating that they might possess potential functions in mammals (see further discussion below).

Besides the reads representing the 9 snoRNA-derived miRNAs, there was a substantial number of reads mapped to either 5′ or 3′ ends of the snoRNAs, exemplified by the reads in blue in Figure 2D (also in File S1). Interestingly, these reads appeared in both Dicer- and Dgcr8-knockout mice data, indicating that they were likely to be the fragments arising from the snoRNA transcripts instead of the miRNAs (Figure S3).

The hairpin structure of sno-miR-#3, as well as that of other snoRNA-derived miRNAs, has additional characteristics. First, it comprises a large asymmetric bulge (4.5 unpaired bases) from the 10- to 13-nt within the miRNA/miRNA* duplex, whereas the seed region is nearly perfectly paired (Figure 2A). Such atypical folding structures were also observed for other known and novel snoRNA-derived miRNAs (Figure S4 and discussion below). Second, the length of the hairpin structure is around 66-nt, similar to the length of a canonical pre-miRNA [28]. Third, the miRNA/miRNA* duplex is located at the end of the hairpin with 2-nt 5′ and 3-nt 3′ overhangs (Figure 2A). In agreement with the observation that snoRNA-derived miRNAs were Dgcr8-inpdependent but Dicer-dependent (Table S3), it is conceivable that the hairpin of sno-miR-#3 mimics the canonical pre-miRNA and thus is suitable for direct Dicer processing after being released from the snoRNA [29].

#### Noncanonical miRNAs derived from tRNAs

We next examined small RNA reads that aligned to the tRNAs in the mouse genome. The well-conserved murine miRNA, miR-1983, derived from murine tRNA-IIeTA [12], yielded 39 reads in our dataset (Table S4). A second tRNA candidate with short hairpin-forming potential, tRNA-SerAGA, was associated with a cluster of 12 small RNA reads (Figure S5A).

The authenticity of this tRNA SerAGA-derived miRNA candidate as a genuine noncanonical miRNA is supported by several observations. First, the tRNA sequence with a 15-nt 3′ extension was predicted to yield an alternative miRNA-like hairpin structure (Figure S5B, right) in addition to the typical tRNA cloverleaf structure (Figure S5B, left). Second, there were mappable sequencing reads corresponding to the two folding structures (Figure S5A), presenting both tRNA fragments and miRNAs. Third, the miRNA was Dgcr8-independent but Dicer1-dependent, as determined by its appearance in the data of Dgcr8-knockout mice but not that of Dicer-knockout mice (Figure S2C and Table S3).

#### miRtrons

A total of 15 candidate new miRtrons, including three typical miRtrons and twelve tailed ones, were identified in the mouse genome (Tables 1 and S3; File S1). They were identified by scanning 160-nt windows covering all intron/exon boundaries in the mouse genome (see Methods). These 15 candidates obeyed both miRNA and miRtron criteria and therefore were considered to be authentic miRtrons. Besides the 15 novel miRtrons, two known miRNAs, miR-702 and miR-5132, should be annotated as a typical miRtron and a tailed miRtron, respectively (Table S4). We also listed in Table S4 the previously annotated miRtrons and the other known noncanonical miRNAs with their digital expression levels in the current small-RNA libraries.

The hairpin of pre-miR-702, which is located in a short intron of a well-conserved mammalian gene *Plod3*, has homogeneous 5′ clustered reads starting with dinucleotide GU and the most abundant 3′ clustered reads ending with dinucleotide AG, which are characteristic of intron splice sites (Figure S6A). Similar observation can be made for miR-5132 hairpin, which is located at the 3′ tail of an intron of the well-conserved mammalian gene *Irak1* (Figure S6B). All the newly annotated miRtrons were Dgcr8-indepdendent but Dicer1-dependent (Table S4).

miRtrons were typically expressed in low abundance in the current sequencing data (the third column of Table 1), which was consistent with the previous observations [10]. Among the novel miRtrons we identified, the most highly expressed was miRtron-#3 with 135 reads, which originated from an intron of gene *Troap*, while miR-1981 (1361 reads, the forth colum of Table S3) was the most abundant among all the miRtrons in our data. Overall, the low abundance of miRtrons may be due to two factors, relatively more unpaired bases in their hairpin structures than canonical miRNAs, and poor conservation in mammals, discussed below.

### Endogenous siRNAs in the mouse genome

**Figure 3:**
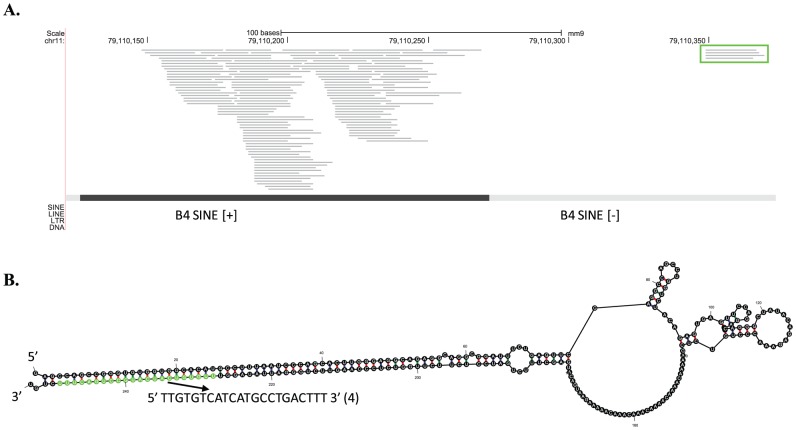
An endo-siRNA derived from tandem inverted B4 SINEs. (A) Alignment of reads to the mouse genome (mm9) and genomic conformation of the two inverted B4 SINEs. The green box indicates the identified siRNAs uniquely mapped to this SINE. The dark black line represents the well-conserved SINE compared to the consensus, while less conserved SINEs are in light gray. The symbol “+” and “−” indicate the orientation of the SINEs. (B) Folding structure of a transcript comprising the two B4 SINEs with the novel endo-siRNA annotated in green.

We searched for putative endo-siRNAs in the sequencing reads originated from putative long hairpin structures in the mouse genome. We identified one novel siRNA locus on chromosome 11 (11qb5) where two tandem B4 SINEs are arranged in a convergent fashion (Figure 3A). Folding of the two SINEs yielded a long hairpin structure comprising a ∼72-nt stem of dsRNAs (Figure 3B). Sequence reads of 21- and 22-nt were uniquely mapped to the stem region of the hairpin and had uniform 5′ homogeneity, characteristic of Dicer-processed small RNAs (File S2). Furthermore, the 22-nt siRNA species was Dgcr8-independent but Dicer1-dependent (Figure S2D).

Besides the newly identified endo-siRNA from B4 SINEs, three previously annotated endo-siRNAs from B1 SINEs in mouse were detected in our virus-infected small-RNA libraries (Table 2). Relative digital expression abundances, in normalized numbers of sequencing reads, of these four endo-siRNAs in the virus-infected cells and in other previously published mouse datasets revealed that these SINE-derived siRNAs expressed more abundantly in murine embryonic stem cells (mESC) and testes and less abundant in newborn-, 7.5-, 9.5- and 12.5-day embryonic cells (Table 2). They were completely absent in ovary and brain [8], [14]. In short, these results suggested a broad existence of SINE-derived siRNAs in various mouse developmental stages and many tissues and cell types.

**Table 2:** Novel and known murine endo-siRNAs represented in 9 sequencing datasets including MHV68-infected (MHV68), mock-treated murine cells (mock; GSE36639); mouse embryonic stem cell (mESC; GSE12521); newborn embryo (nb), 7.5-, 9.5-, and 12.5- day point embryo cells (e7p5 etc.), testes (GSE20384) and oocyte (GSE10364).

ID	sequence	MHV68	mock	mESC	nb	e7p5	e9p5	e12p5	testes	oocyte
siRNA3^a^	UUGUGUCAUCAUGCCUGACUUU	4	0	43	0	7	0	0	0	0
siRNA1.1^b^	AAGCCGGGCCGUAGUGGCGCA	14	4	2051	0	78	8	43	25	0
siRNA1.2^b^	AGCCUUUAAUUUCAGUACUUGG	26	9	14105	52	7	267	8	25	0
siRNA2.1^b^	CCUUUAAUCUCGACACUUGGA	5	0	175	0	70	142	43	1574	21

Read counts were normalized across the samples.

a newly identified siRNA.

b siRNAs annotated in the previous study [12].

### Conservation of novel miRNAs and endo-siRNAs in the animal kingdom

We studied the conservation of the novel miRNAs and endo-siRNAs via a comparative genomics analysis across seven species in mammals. As listed in Table S6, a total of 30 out of 47 (63.8%) novel miRNAs and all of the four endo-siRNAs in mouse were only recognized in *Mus Musculus* (mouse) or *Rattus norvegicus* (rat). In addition, six out of nine snoRNA-derived, the tRNA-derived, and four out of 22 canonical miRNAs, as well as six out of 15 miRtrons were found conserved beyond Rat and further down to *Monodelphis domestica* (opossum), exemplified by the well-conserved sno-miR-#1 and sno-miR-#9. As for the endo-siRNAs, the three B1 SINE-derived siRNAs appeared only in mouse, while the newly identified B4 SINE-derived siRNA was conserved in rat (Table S6). This might largely result from the conservation of the repeat elements hosting the endo-siRNAs. For instance, B1 and B4 SINEs were found to be *Mus Musculus* and Rodentia specific, respectively (RepBase version16.11) [30], [31]. In sum, the majority of the miRNAs and endo-siRNAs were not conserved beyond Rodentia, suggesting that they were newly evolved and might have rodent-specific functions. We did not find any homologs of the two new viral miRNAs in EBV and KSHV genomes, which is in agreement with the previous work studying the miRNAs conservation in herpesvirus [32]. Besides, we have examined the expression of these sequence-conserved miRNAs in deep sequencing data from human psoriatic and normal skin [33]. Of 17 conserved miRNAs in mammals, 7 were detected in human skin. The complete result of the sncRNAs conservation was provided in File S3.

### Distinctive features of noncanonical miRNAs

Making use of all the noncanonical miRNAs we identified and detected, we were able to study their sequence and structural features. Indeed, they have several characteristics, making them distinct from the canonical miRNAs.

#### Short hairpins of noncanonical miRNAs

**Figure 4:**
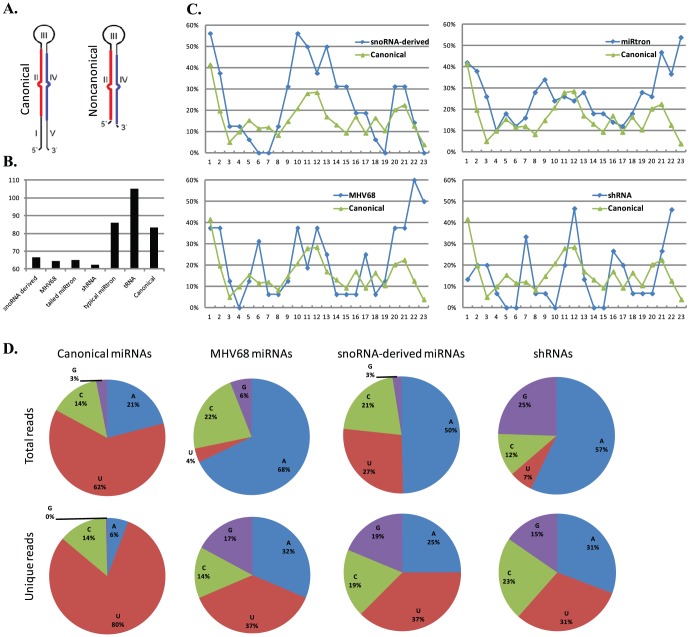
Features of canonical and noncanonical miRNAs. (A) A diagram marks the locations of the five regions for canonical miRNA hairpins: I, loop-distal (5′ arm); II, miRNA-5p; III, loop-proximal; IV, miRNA-3p; V, loop-distal (3′ arm). Noncanonical miRNA hairpins miss I and V loop-distal regions. (B) The average length of hairpins for canonical miRNAs, snoRNA-, tRNA-derived miRNAs, miRtrons (typical and tailed) and MHV68-encoded miRNAs (C) The percentage of the number of unpaired bases at each position (from 5′ to 3′) for canonical (green) and noncanonical (blue) miRNAs, respectively. (D) First nucleotide percentage of total reads (upper) in MHV68 data mapped to conserved, MHV68 encoded, snoRNA-derived miRNAs and shRNAs. Percentages of unique miRNAs are presented in the lower part, respectively.

We observed that all the noncanonical miRNA hairpins were missing 5′ and 3′ arm loop-distal regions, marked as I and V regions of a canonical miRNA hairpin (the foldback structure before Drosha and Dgcr8 processing) in Figure 4A, thus making all the noncanonical miRNA/miRNA* duplexes locate at the hairpin ends with potential 1- to 3-nt 3′ overhangs. Second, the average length of most noncanonical miRNA hairpins was 64.5-nt, in agreement with the average length of canonical pre-miRNAs (foldback structures after Drosha processing) [28], but in contrast to the length of canonical miRNA hairpins (83.3-nt, Figure 4B). Two exceptions arise from typical miRtron and tRNA-derived miRNA hairpins, which were 86.1-nt and 105-nt long on average, respectively (Figure 4B). However, the longer hairpins of typical miRtrons and tRNA-derived miRNAs resulted from their prolonged loop-proximal regions, *i.e.*, the miRNA duplexes were still located at the hairpin ends, different from the canonical miRNA hairpins with loop-distal regions.

#### Atypical base paring

Noncanonical miRNAs have atypical base pairings that appear more frequently in the central regions from 9- to 14-nt or near both ends within miRNA duplexes (Figure 4C). The most prominent examples appeared in snoRNA-derived miRNAs and miRtrons, which have average 5.4 unpaired bases compared to 3.6 for well-conserved canonical miRNAs (Figure 4C, upper two panels). The extra unpaired bases, which averaged at 1.8-nt, occur more frequently on the 5′ first nucleotides, in the central regions or near the 3′ ends (21- to 22-nt) of miRNA/miRNA* duplexes; whereas the seed regions (2- to 8-nt) have a high complementarity along with their pairing partners (15- to 19-nt) on the other arms of the hairpins (Figure 4C, upper panel). Similar patterns were also observed on MHV68-encoded miRNAs and shRNAs (Figure 4C, lower left panel).

#### Altered first nucleotide preference

A large portion of noncanonical miRNAs have Adenine or Cytosine as the first nucleotides, exemplified by the MHV68 miRNAs with ∼90% of the total reads starting with A or C (Figure 4D upper panel). The unique sequences of MHV68 miRNAs also have a higher A and C preference (45%) than unique sequences of canonical miRNAs (20%). As a comparison, such preference for A and C as the first nucleotides were quite different from the well-known preference of Uracil for canonical miRNAs. Indeed, about 73% of the total reads mapped to the conserved canonical murine miRNAs start with U in our small-RNA libraries. Likewise, about 86% of the total reads mapped to the miRNAs in the KSHV genome also start with U [34]. It is worthwhile to note that many canonical miRNAs with non-U first nucleotide have been shown to have important functions [35]–[37].

### Viral and viral responsive sncRNAs are potentially functional

Small noncoding RNAs exert their function via regulation of their target, mostly protein-coding genes. Of particular interest are those sncRNAs that are responsive to viral infection and their corresponding targets.

#### Expressed viral miRNAs and murine miRNA responsive to MHV68 infection

The two newly identified and the 15 known MHV68 miRNAs were all expressed in virus-infected cells, implying their potential regulatory functions during lytic infection. More importantly, one of the murine miRNAs (miR-142-3p) in the host genome exhibited more than 35-fold up-regulation in lytically infected cells in reference to mock-treated cells with the percentage of false positive (pfp) less than 0.001 (see Methods).

#### Targets of viral and viral responsive miRNAs

Expressed viral miRNAs have the potential to target transcripts in the virus and/or the host to exert their functions. To appreciate their potential regulatory roles, we searched for putative targets of virus miRNAs and isomiRs (see Methods). This resulted in 26 ORFs in the MHV68 genome and 4653 host protein-coding genes to be potentially targeted by all 17 MHV68 miRNAs (Tables S7 and S8). Among the MHV68 transcripts targeted by the virus miRNAs, ORF M2 was found to be potentially targeted by two cognate MHV68 miRNAs, miR-M1-8 and miR-M1-13, both of which are located antisense to M2 and thus perfectly match to M2 sequence with a perfect reverse complementarity. In contrast to a relatively small number of targets in the viral genome, a large number of genes in mouse might be targeted by the virus miRNAs, suggesting that a large cassette of host genes could be manipulated by viral miRNA genes through miRNA-guided mRNA regulation. Interestingly, among the mouse targets, there were eight genes that had been previously detected down-regulated in MVH68-infected cells [38]. The anti-correlated expression between the MHV68 miRNAs and the mouse genes might indicate the interactions between the virus and the host.

Furthermore, the significance of the differential expression of mouse miRNA, miR-142-3p, suggests involvement in gene regulation during lytic infection of MHV68; thus its targets are of primary interest for further investigation. A total of 240 mRNA genes in the mouse genome were predicted to be putative targets of miR-142-3p (Table S9). On the other hand, no ORF in the viral genome was predicted to be a target of miR-142-3p, indicating that MVH68 might have evolved to avoid counter-attack of the host miRNAs' regulation.

#### Function enrichment of putative targets of miRNAs

The large number of putative targets made it possible to further analyze which cellular and signaling pathways may be potentially regulated by the viral and viral-responsive miRNAs. We thus examined Gene Ontology (GO) terms enriched in the putative target genes identified. Among many biological processes revealed by the GO analysis were transcription and protein amino acid phosphorylation (Table S10). These basic regulatory functions indicated that the viral and viral-responsive miRNAs have the potential to alter transcription of some of the genes in the host and influence protein phosphorylation that activates or volatizes host proteins, so as to plausibly escape the defense of the host. Many of these target genes were localized to cell membrane (Table S10), where most signaling receptors function. These results implied that the viral and viral-responsive miRNAs detected and analyzed in the current study might possibly be involved in basal immune response during MHV68 lytic infection.

## Discussion

Phenotypic differences between Dicer-null and Drosha-null mutants have been observed in recent studies, which implied a potential role for noncanonical miRNAs and endo-siRNAs [39]. For example, Dgcr8-null mutant has less severe phenotype than Dicer-null mutant in mouse embryonic cell [12]. Such phenotypic differences in part motivated the current study to look for noncanonical miRNAs and endo-siRNAs. To the best of our knowledge, little has been done to systematically explore novel noncanonical miRNAs and endo-siRNAs from various genomic origins in animal organisms. Next-generation sequencing has made it feasible to expand the research of these small-RNA species to an unprecedented level. In this study, we carried out a whole-genome, systematic search of novel canonical and noncanonical miRNAs as well as endo-siRNAs in MHV68-infected murine cells. Meanwhile, we developed a computational approach and software pipeline that effectively utilizes various small-RNA profiling data, through which we could not only identify novel miRNAs and endo-siRNAs but also analyze their characteristics. Our results revealed several distinct features of noncanonical miRNAs and showed murine MHV68 infection could induce endo-siRNAs from SINEs murine cells (Table 2).

### 

#### Virus miRNAs

We identified two novel miRNAs that reside antisense to two known miRNAs in MHV68. Antisense miRNAs have been previously reported in human, Drosophila, KSHV and herpes simplex virus type1 (HSV-1) [34], [40], [41] and their functional role in gene regulation has been analyzed in Drosophila [41]. The generation of an antisense miRNA could result from bidirectional transcription of a miRNA locus. When the antisense transcript has the capacity to fold into a hairpin structure, it could potentially undergo the miRNA biogenesis pathway. In most cases, antisense miRNAs are expressed at lower abundance than their sense counterparts, as shown in the current analysis and previous studies [34], which indicated that it is possible that antisense miRNAs might be byproducts of the sense miRNAs. Furthermore, most sense and antisense miRNAs share high sequence similarities and thus have potentially similar functions like the well-studied example in Drosophila [41].

We observed a substantial amount of sequence variations of virus miRNAs and recognized that one third of virus miRNAs had 5′ heterogeneous isoforms or isomiRs. Some of the isomiRs had comparable abundances as the major miRNAs. This is crucial because 5′ heterogeneity could shift miRNA seed regions and thus alter their downstream target binding. The large amount of miRNA isoforms that we observed may due to the different cells and conditions used in our and previous experiments. The annotation of some of the MHV68 miRNAs in miRBase was based on sequencing data of MHV68-infected murine NIH3T3 and S11 cells [19]. Such discrepancies may suggest plasticity of miRNA gene regulation under different conditions.

#### Noncanonical miRNAs in mice

It is surprising that the mouse genome encodes so many noncanonical miRNAs in loci of a variety of small noncoding RNAs (sncRNA), including snoRNAs and tRNAs, through alternative RNA folding of cognate transcripts. The flexibility of producing miRNAs from such sncRNAs implies the potential of cognate sncRNA transcripts to derive alternative regulatory functions. Indeed, a murine ACA45-derived miRNA has been recently shown to share seed region similarities with canonical miRNAs, which implied a regulatory role of gene regulation [14]. Besides, while the maturity of the snoRNA-derived miRNAs are not Drosha dependent, the microprocessor substituting Drosha for releasing these miRNA precursors remains unknown [42]. Moreover, the determination of which alternative folding structure is dominant seemed to be tissue specific, exemplified by the tRNA-derived miRNAs in our study and in the previous study [12]. Moreover, presence of a set of species-specific miRNAs derived from near intron-exon boundaries suggested potential function for the debranched introns in gene regulatory network.

Besides those pre-miRNAs piggybacking on other annotated sncRNA transcripts, intergenic shRNAs originated from unannotated transcripts provided more examples that pre-miRNAs are broadly encoded in mammalian genomes. We also observed that individual members of single shRNA family tend to follow the same biogenesis pathway. For example, the members of miR-344 family were all produced from shRNAs (Table S4).

#### Features of noncanonical miRNAs

Distinct from canonical miRNAs, most noncanonical miRNAs shared some common features of their hairpin structures, lengths, base pairings and first nucleotide preferences. All the noncanonical miRNA hairpins that we analyzed were observed to lack distal-loop regions, thus, having an average length of 64.5-nt. Such a structural property is in agreement with the observation that noncanonical miRNA hairpins are similar to pre-miRNAs.

Typical miRtrons and tRNA-derived miRNAs tend to have longer hairpin structures, which can give rise to two adjacent miRNA species. One example, miRtron miR-3102 precursor, is 104-nt long and can generate two distinct miRNA/miRNA* duplexes, namely outer (close to the basal end) and inner miRNAs (close to the loop end) [8]. Such long pre-miRNA structures were also observed for miR-702 precursor (102-nt) and two tRNA-derived miRNA precursors (average 105-nt) in our study (Figure S5 and File S1). However, no sequence reads were found in all the data for the inner miRNAs derived from miR-702 and tRNA-derived miRNA precursors, which might be due to weak base pairings within these inside sequences that curdle a second Dicer activity. It is noteworthy that long pre-miRNAs in plants [43] and viruses [44] may host multiple miRNAs.

More unpaired bases appeared in snoRNA-derived miRNAs and miRtrons than well-conserved canonical miRNAs. Nonetheless, instead of being inside seed regions, the extra unpaired bases occurred more often at the first nucleotides, in the central regions and near the 3′ ends of miRNAs (Figure 4B). Such pattern of base pairing indicated a broader form of substrates cleaved by Dicer, which only requires correct base pairings in the seed regions but can tolerate weak base pairings in the central regions or at the two ends of miRNAs. Alternatively, one or two unpaired bases in the seed regions might be compensated by the overall correct base pairings. This is in agreement with the observation that noncanonical miRNA hairpins can be effectively processed by Dicer. Finally, a preference for A and C as the first nucleotide was observed for noncanonical miRNAs, which is a significant deviation from the well-documented preference of U for canonical miRNAs.

#### Plausible functions of noncanonical viral and murine miRNAs in lytic infection

Constructed at a particular time during MHV68 lytic infection, the five small-RNA libraries that we analyzed in the current study may be too small to draw a concrete conclusion on the functions of noncanonical miRNAs. Despite this limitation, the data and results, particularly those on the targets of viral miRNAs and the differentially expressed murine miRNA, offered a glimpse into the possible functional roles they may play. As listed in Table S10, the three main biological processes enriched in the set of target genes of these miRNAs are transcription, regulation of transcription and protein phosphorylation. This result indicated that these miRNAs were directly involved in regulating gene transcription and protein modification that affect which state, active or inactive, that these proteins might be in. Furthermore, among the six significant cellular components that these target genes may locate were three components related to cell membranes (Table S10), where many signaling receptors reside, which alluded to plausible involvement of these miRNAs in regulating signaling transduction during lytic infection. In addition, the top five molecular functions that these target genes are associated with were all exclusively related to various ion binding, including metal ion binding (Table S10). As reviewed by Johnson-Buck et al. [45], it has been recognized that metal ions are essential for the proper folding and function of small ribozymes, which in turn, play vital roles in the replication of viral pathogens. In sum, all these results on the functions of miRNA target genes implied miRNAs' possible involvement and function in basal immune response during MHV68 lytic infection.

#### Murine endo-siRNAs

We identified one locus comprising two tandem B4 SINEs to yield a long hairpin and further to give rise to endo-siRNAs. These endo-siRNAs were enzymatically Dgcr8-independent but Dicer-dependent, as expected for endo-siRNAs. Previous studies also revealed a number of examples of endo-siRNAs derived from either B1 SINEs or long terminal retrotransposons (LTRs) in mouse stem cells or oocytes [23], [24], respectively. All these loci encode inverted repeats that can host long hairpin structures. Endo-siRNAs derived from these loci were usually arranged in a 21- to 22-nt phasing fashion.

endo-siRNAs were presumed to be largely absent from mammalian cells, a notion bolstered by the assumption that long double stranded RNAs (dsRNAs), a rich source for siRNA generation, would trigger an interferon (IFN) response [46]. Surprisingly, a sufficient number of SINEs-derived endo-siRNAs were observed in the current virus-infected murine deep sequencing data where the IFN might be triggered by virus infection [47]. This raises the question of how these long dsRNAs derived from SINE elements could avoid the surveillance of mammalian adaptive immune systems, which are supposed to recognize and degrade long dsRNAs. One possibility is that dsRNAs derived from SINE hairpin structures are shorter compared to those dsRNAs generated in virus infection or have other unknown features that distinguish themselves. Interestingly, we also observed SINE-derived endo-siRNAs in a variety of published sequencing data of various tissues and conditions; however, none were found to be derived by LTRs.

endo-siRNAs have been reported to originate from other diverse sources including cis-NATs, trans-NATs and Piwi-associating loci previously [23]. The most prominent examples are cis-nat-siRNAs derived from annealed natural antisense transcripts (NATs). Hybridization of NATs yields long dsRNAs followed by Dicer processing to derive siRNAs which regulate targeted transcripts. When mapping sequence reads in MHV68-infected small-RNA data to the 17 annotated cis-nat-siRNA-annotated loci in the mouse genome, six had a sufficient number of reads in the overlapping regions of the cis-NATs (Figure S7). However, the somewhat random length distributions of the total mappable reads in these regions require more experimental data in order to correctly categorize these small-RNA reads.

#### Computational identification of noncanonical miRNAs and endo-siRNAs

Identification of noncanonical miRNAs and endo-siRNAs can be difficult due to the plausible presence of heterogeneous small RNAs originating from the same genomic loci, as shown by the snoRNA- and tRNA-derived miRNAs as well as SINE-derived siRNAs. Additionally, atypical folding of corresponding transcripts complicated the discovery procedure. To increase the sensitivity of the procedure, we proposed an integrative computational method by considering multiple features of these small RNAs. The examination of the enzymatic dependency of both individual sequence reads and clustered aligned reads helped differentiate small RNAs derived from different origins. Furthermore, the presence of sequence reads characteristic of Dicer cleavage provided additional information for accurate identification. Such characteristics included the length distribution, 5′ or 3′ end homogeneity, characteristic 5′ or 3′ end of clustered reads that we explored in our study.

## Methods

### Samples and small RNA library preparation and sequencing

Total RNA from mock- and MHV68-infected NIH 3T12 cells were prepared as previously described [18]. Total RNA libraries for sequencing were prepared using the small RNA sample prep kit (Illumina). Twenty micrograms of total RNA were used as input, and small RNAs were size selected by running total RNA on a 15% denaturing polyacrylamide gel and cutting out the RNA corresponding to 18- to 30-nt. The libraries were sequenced using Illumina GAII.

### Read processing and mapping

Individual deep sequencing libraries were each processed separately as follows. Reads containing no sequencing adaptor sequence, reads having a substring of adapter strictly shorter than 6-nt, or reads whose trimmed sequences were shorter than 17-nt were removed from further analysis. Adaptor-trimmed reads were mapped to the MHV68 [48] (NC_001826.2) and mouse genome sequences (UCSC mouse genome [49], mm9 build) by Bowtie [50] and sequences in several noncoding and small RNA databases, specifically UCSC genome browser tables (tRNA track), microRNA (miRBase version17, http://www.mirbase.org/ftp.shtml), murine snoRNA (Ensembl noncoding genes, version64) and repeats regions (UCSC RepeatMasker Table, mm9 build).

### Identification of noncanonical miRNAs

The qualified reads were mapped to the known miRNA loci by Bowtie [50] and any matching reads were considered as fragments originating from known miRNA loci and thus were discarded. The remaining qualified reads aligned to mouse snoRNAs and tRNAs were subjected to our miRNA prediction method. For each snoRNA and tRNA, 50-nt extensions on both ends of the transcript were added for secondary structure analysis. In order to find the most proper hairpins, multiple secondary structures were predicted by RNALfold [51]. Predicted structures lacking stems of at least 18-nt or lacking more than 10 reads mapped to the stems were excluded. The flowchart of the steps of miRNAs identification is provided in Figure S1. The minimum copy number of 10 reads was empirically determined by considering the percentage of the total genomic-aligned reads. Figure S8 shows the relationship between the minimum copy number used and the percentage of reads included. More than 99% of reads will be included by choosing the cutoff of 10 reads.

Candidate miRNAs were prioritized based on (1) occurrence of sequencing reads on the stem of a predicted hairpin structure with minimum folding energy less than -18 kcal/mol [33]; (2) possible presence of miRNA* reads on the opposite stem of the hairpin; (3) presence of a ∼2-nt 3′ overhang on the highest likelihood miRNA/miRNA* duplex; (4) reads length distribution peaks at ∼22-nt; and (5) Dicer dependency if dicer-knockout data were available, particularly when no miRNA* sequence was detected.

To identify miRtrons, a 160-nt intronic sequence adjacent to exon/intron boundary was extracted for each intron in the mouse genome (RefSeq Table; UCSC genome browser; mm9 build). The same procedure for finding miRNAs as described above was then applied to identify miRtrons. In addition, the presence of sequencing reads starting or ending at intron splice sites was examined as a signal of miRtrons. The suboptimal secondary structures within an folding energy difference of 5 kcal/mol predicted by RNAsubopt [51] were subsequently inspected visually.

### Identification of endogenous siRNAs

The EINVERTED program [52] was applied to each 10-kb segment of the whole mouse genome (mm9 build) and cDNA sequences (Ensembl version 64) and a minimum score threshold of 70 [53] was used in the program to find long and well base-paired dsRNAs different from short or loosely annealed hairpins. This resulted in more than 550 K long dsRNAs in the mouse genome and cDNA sequences. Long dsRNAs with more than ten unique genome mappable reads were considered siRNA-deriving candidate loci for further analysis.

To identify authentic endo-siRNAs derived from long dsRNAs, we calculated the length distribution of reads uniquely mapped to each candidate and required that more than 60% of the mappable reads must be 21- to 23-nt long, as typically expected for siRNAs [23], [54]. Next, we plotted the reads alignment for each candidate long dsRNA which satisfied the length distribution criterion. Based on the alignment, we visually searched for a locus on which clustered reads had a clear 5′-end homogeneity, as this reflects Dicer cleavage activities. Furthermore, we also examined the Dicer dependence and Dgcr8 independence of the reads originating from the examined locus in the rich collection of small-RNA sequencing data from embryonic stem cell (GEO accession no. GSE12521). The authentic siRNAs were expected to be those reads that did not appear in Dicer-knockout data but appeared abundantly in Dgcr8-knockout data. Finally, we classified the most abundant candidate reads that satisfied all the criteria as genuine siRNAs.

### Conservation of sncRNAs

To analyze conservation of sncRNAs, we retrieved from the UCSC genome browser [55] multiple alignments of eight species – *Mus musculus* (mouse), *Rattus norvegicus* (rat), *Homo sapiens* (human), *Pongo pygmaeus abelii* (Orangutan), *Canis lupus familiaris* (dog), *Equus caballus* (horse), *Monodelphis domestica* (opossum), and *Gallus gallus* (chicken) – for each novel sncRNA. Next, based on the multiple alignments of the eight species, we calculated the number of insertions, deletions and mismatches when each non-murine sequence was compared to the murine sncRNA (File S3). An sncRNA was considered conserved in one species if the sequence in that species had no more than two variations (insertions, deletions and mismatches) in the seed region for a miRNA or the whole region for a siRNA.

### Characteristics of noncanonical miRNAs

To calculate the average length of canonical miRNAs, we fetched the hairpin structures of well-conserved miRNAs from miRBase (version 17). The hairpin sequences of novel and newly annotated noncanonical miRNAs, provided in File S1, were determined by the folding structures (from RNALfold) of genomic loci encompassing sequencing reads corresponding to miRNA candidates. The number of unpaired bases in miRNA/miRNA* duplexes was calculated based on the folding structure. We only considered mature miRNA sequences when calculating the number of unpaired bases since mature miRNAs were the most representative regions. To evaluate the 5′ first nucleotide preference for noncanonical miRNAs in various cells and conditions, we calculated the percentage in a series of sequence data including mouse embryonic stem cell (GSE12521); newborn embryo, 7.5-, 9.5-, and 12.5- day point embryo cells (e7p5 etc.), ovary, testes, brain (GSE20384); oocyte (GSE10364); and cortex and hippocampus (GSE21090).

### Differential expression of sncRNAs

We collected the newly identified sncRNAs and the known murine miRNAs (miRBase version 17) for differential expression analysis. Reads aligned perfectly to the set of sncRNAs with 3-nt extension on either end, in order to include their possible isoforms, were considered in the analysis. Reads mapped to multiple genomic loci were attributed to all derivative small RNAs. Read counts in each sample were normalized to adjust for variation in total read count between samples. Let N_sample_ be the number of qualified reads that aligned to the mouse genome (mm9 build) in each sample, C the average of all N_sample_, and M_sample_ the number of qualified reads in each sample aligned to each sncRNA. Thus, the normalized number of reads for each sncRNA in a given sample is (M_sample_*C)/(N_sample_). Fold changes were calculated from the average normalized read counts in each category (MHV68-infected or mock-treated). We identified differentially expressed genes using Rank Product (RP) implemented in R [56]. It has been shown that RP is less sensitive to noise and has a better performance than other methods when sample size is small [57]. We selected differentially expressed genes with the percentage of false positive rate (pfp) no greater than 5% for 1000 permutations.

### Targets of miRNAs

Sequences for MHV68 ORFs were downloaded from NCBI website (NC_001826.2). Sequences for host murine 3′ UTRs were fetched from the Refseq table in UCSC genome browser (mm9 build). The two sets of sequences were then combined as a whole for target prediction. We applied miRanda [58] to the miRNAs of interest, *i.e.*, virus miRNAs and differentially expressed host miRNAs, to find their putative targets. We set a minimum length cutoff greater than 160-nt and no mismatches in miRNA seed regions to predict targets.

### GO term and Functional enrichment of target genes

The GO term analysis was performed using the online tool DAVID [59]. DAVID gives a *p*-value, before and after multiple-test corrections, based on a modified Fisher's exact test.

### Data deposition

All small-RNA deep-sequencing data have been deposited into NCBI/GEO databases, and the accession number is GSE36639.

## Supporting Information

Figure S1 **Flowchart describing the major steps for novel miRNAs identification.**
(PDF)

Figure S2 **Normalized reads in wide-type, Dicer- and Dgcr8-knockout mice (GSE12521).** (A) snoRNA-derived miRNA candidate #3. (B) snoRNA-derived miRNA candidate #9. (**C**) tRNA-derived miRNA discussed in the main text. (**D**) endo-siRNA derived from inverted B4 SINEs.
(PDF)

Figure S3 **Sequence reads aligned to sno-miR-#3.** Reads corresponding to snoRNA fragments (in blue) appeared in both Dgcr8- and Dicer-knockout mice, while representative miRNA reads were found in Dgcr8- but not in Dicer-knockout mice.
(PDF)

Figure S4 **Examples of known and novel snoRNA-derived miRNAs bearing atypical folding structures.** (A) Folding structure of snoRNA HBI-100 carrying an annotated miRNA,mmu-miR-1843 with 4.5 unpaired bases in the central bulge, indicated inside the red ellipsis. (B) Folding structure of SNORA1 carrying novel sno-miR-#9 with 5 unpaired bases in the central bulges.
(PDF)

Figure S5 **A murine miRNA derived from tRNA-SerAGA.** (A) Alignment of reads representing the tRNA fragments and miRNA sequences. The tRNA is annotated in blue with the putative Pol III transcription terminal signal (in orange). Untemplated reads derived from tRNA CCA addition end with blue characters, while miRNA reads are annotated in red. The arrows indicate the RNase P (left) and RNase Z (right) processing. (B) The tRNA folding structure (left) and the alternative miRNA hairpin structure (right) with the miRNA annotated in red, with folding energy being presented below each structure. (C) Multiple sequences alignments of tRNA-SerAGA sequence among eight mammalian species with the miRNA indicated in the red rectangle.
(PDF)

Figure S6 **Sequence reads mapped to the (A) mmu-miR-702 and (B) mmu-miR-5132 hairpins.** Sequence in blue represents intron region, while in yellow represents flanking exon regions. Reads comprising GU or AG dinucleotide, which is characteristic of intron splice sites, were marked in red.
(PDF)

Figure S7 **Six of 17 previously annotated cis-NATs in the previous study** [**24**] **appeared in the current MHV68-infected data.** All of the loci are based on UCSC mouse reference genome (version mm8). Mapping of reads and numbers of reads (log2 based) in current data set (GSE36639) and in oocyte (GSM261957) are shown in the top two tracks. RefSeq and Ensembl Gene annotations, repeat elements and conservation scores are shown (see USCS genome browser for details of track information).
(PDF)

Figure S8 **The relationship between the copy number and the percentage of all genomic-aligned reads included.**
(PDF)

File S1 **Read alignments for 49 novel miRNAs in** **Table 1** **and Table S4.** The first column on the left indicates the number of reads in the sequencing data; the second column indicates the length of the read; the third column indicates how many loci in the MHV68 or mouse genome (version mm9) the read can be mapped to.
(TXT)

File S2 **Read alignments for the novel and previously annotated murine endo-siRNA in current sequencing data.** The genome information was based on UCSC mouse genome annotation (mm9 build).
(TXT)

File S3 **Multi-species alignments of sncRNAs among seven species.** The number of insertion (ins), deletion (del) and mismatches (mis) compared to mouse sncRNAs are listed. The multi-species include mouse (mm9), rat (rn4), human (hg18), orangutan (ponAbe2), dog (canFam2), horse (equCab1) and opossum (monDom4). Small RNAs from the sequencing data of human psoriatic skin (GSE31037) were aligned to human homologous sequences.
(TXT)

Table S1 **Reads mapped to the known miRNAs (miRbase version17).**
(XLSX)

Table S2 Abundance of MHV68 miRNA, miRNA* and their isoforms. The sequences underlined indicate the most abundant read. The annotated miRNA sequences (miRbase version 17) were indicated in column miRbase. Five miRNAs showing 5′ isoforms are marked in yellow.
(XLSX)

Table S3 **Twenty-eight novel miRNAs in the mouse genome represented by normalized reads from wide-type (wt), Dicer- (dicer) and Dgcr8-knockout (dgcr8) mice** [**12**] **.**
(XLSX)

Table S4 **Newly annotated and previously described noncanonical miRNAs.** The column of # Reads represents the number of reads in the current sequencing data for each mature miRNA sequence. Normalized reads from wide-type (wt), Dicer- (dicer) and Dgcr8-knockout (dgcr8) mice [12] were included.
(XLSX)

Table S5 **A set of miRNA candidates identified in the mouse genome (build mm9).** The columns of # reads represent the number of reads in all samples perfectly mapping to mature miRNA and hairpin sequences, respectively. The column of location indicates the names of host genes overlapping with miRNAs.
(XLSX)

Table S6 **Conservation of noncanonical miRNAs and endo-siRNAs that were analyzed.**
(XLSX)

Table S7 **MHV68 open reading frames targeted by MHV68 virus miRNAs.** The number at the end of the miRNA name represents the digital counts of the miRNA and indicates the corresponding isoform. Annotation of MHV68 open reading frame can be downloaded from NCBI with accession number NC_001826.2.
(XLSX)

Table S8 **List of genes in mouse genome targeted by MHV68 virus miRNAs.**
(XLSX)

Table S9 **List of genes in mouse genome targeted by the differentially expressed murine miRNA (miR-142-3p).**
(XLSX)

Table S10 **Representative Gene Ontology functions of predicted genes targeted by virus and differentially expressed miRNAs.**
(XLSX)
