# Supplementary figures and images for: Noncanonical MicroRNAs and Endogenous siRNAs in Lytic Infection of Murine Gammaherpesvirus
Source: PLoS One. 2012 Oct 26;7(10):e47863. doi: 10.1371/journal.pone.0047863 (PMC3482243; doi:10.1371/journal.pone.0047863)

Supplemental Figure S1. Flowchart describing the major steps for novel miRNAs identification.

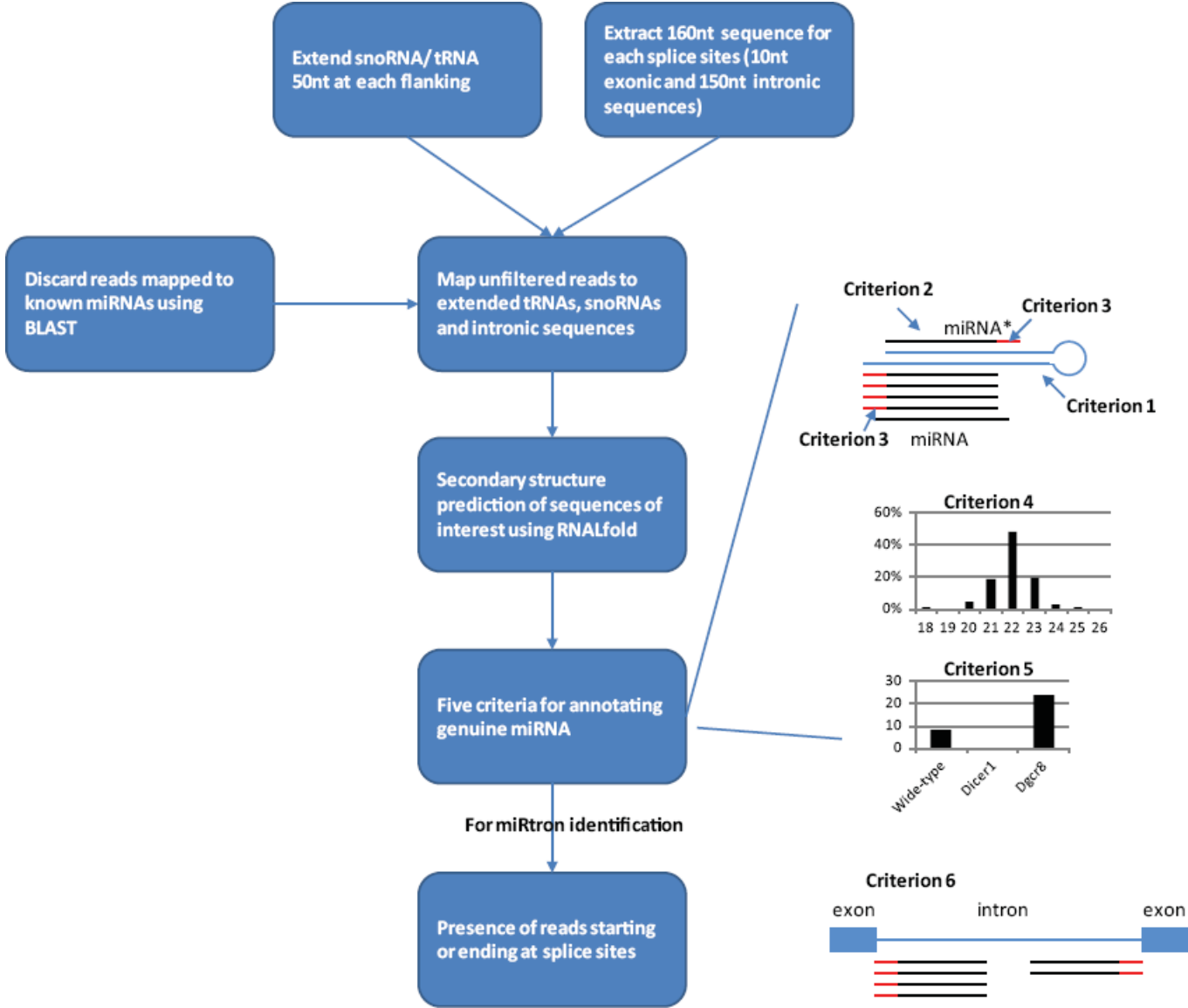

Supplement: Figure S1 — Flowchart describing the major steps for novel miRNAs identification. (PDF) [file pone.0047863.s001.pdf]
