# Supplementary material for: Noncanonical MicroRNAs and Endogenous siRNAs in Lytic Infection of Murine Gammaherpesvirus
Source: PLoS One. 2012 Oct 26;7(10):e47863. doi: 10.1371/journal.pone.0047863 (PMC3482243; doi:10.1371/journal.pone.0047863)

Supplemental Figure S2. Normalized reads in wide-type, Dicer- and Dgcr8-knockout mice (GSE12521). (**A**) snoRNA-derived miRNA candidate #3. (**B**) snoRNA-derived miRNA candidate #9. (**C**) tRNA-derived miRNA discussed in the main text. (**D**) endo-siRNA derived from inverted B4 SINEs.

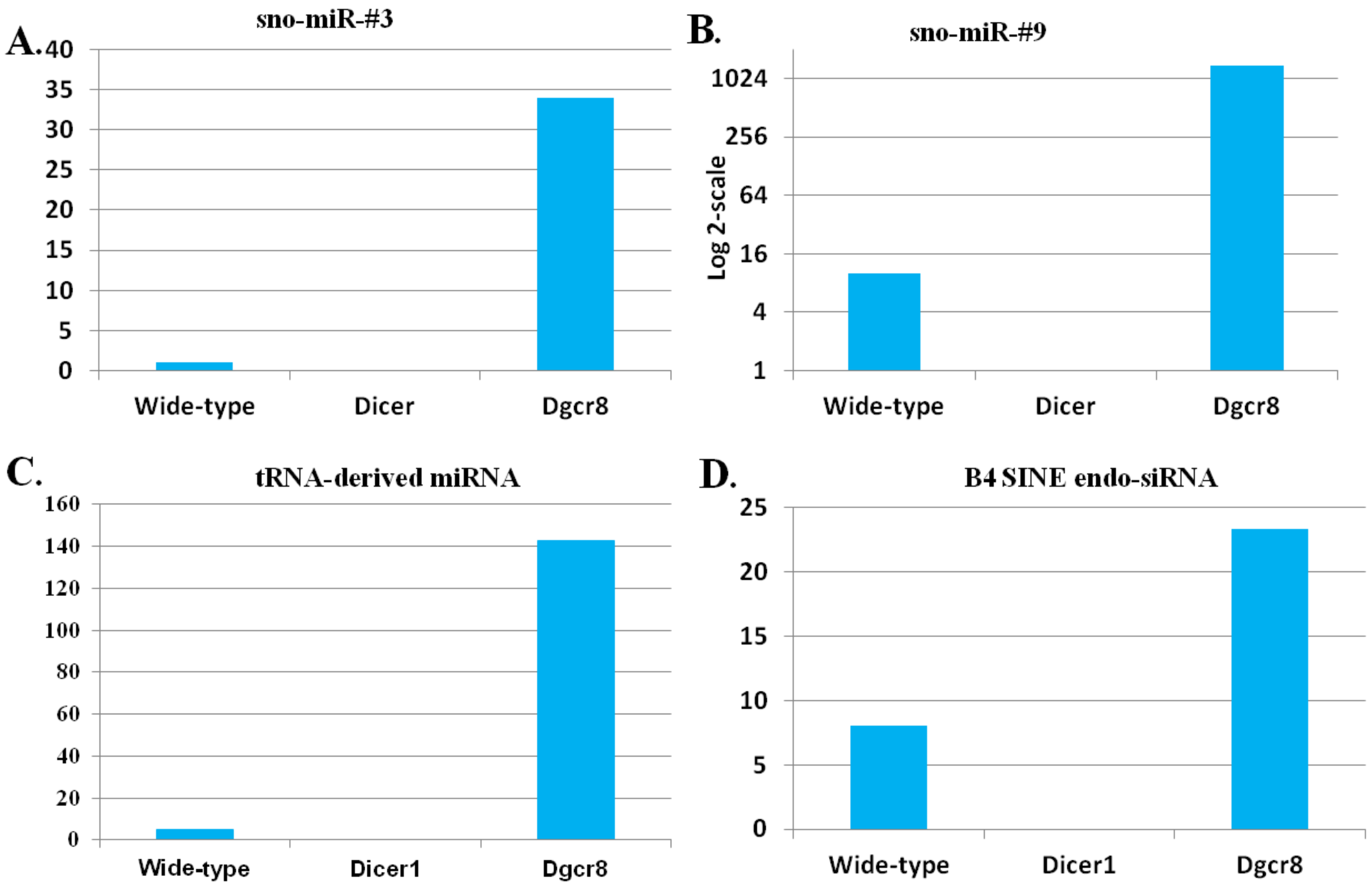

Supplement: Figure S2 — Normalized reads in wide-type, Dicer- and Dgcr8-knockout mice (GSE12521). (A) snoRNA-derived miRNA candidate #3. (B) snoRNA-derived miRNA candidate #9. (C) tRNA-derived miRNA discussed in the main text. (D) endo-siRNA derived from inverted B4 SINEs. (PDF) [file pone.0047863.s002.pdf]
