# Supplementary material for: Noncanonical MicroRNAs and Endogenous siRNAs in Lytic Infection of Murine Gammaherpesvirus
Source: PLoS One. 2012 Oct 26;7(10):e47863. doi: 10.1371/journal.pone.0047863 (PMC3482243; doi:10.1371/journal.pone.0047863)
Supplement: Figure S3 — Sequence reads aligned to sno-miR-#3. Reads corresponding to snoRNA fragments (in blue) appeared in both Dgcr8- and Dicer-knockout mice, while representative miRNA reads were found in Dgcr8- but not in Dicer-knockout mice. (PDF) [file pone.0047863.s003.pdf]

Supplemental Figure S3. Sequence reads aligned to sno-miR-#3. Reads corresponding to snoRNA fragments (in blue) appeared in both Dgcr8- and Dicer-knockout mice, while representative miRNA reads were found in Dgcr8- but not in Dicer-knockout mice.

sno-miR-#3

```
GACCUCUUGGGAUCGCGUCUGGAGAGUAGCUAGUAUUCUUCCAGCUUCGGAAGCAAGAGGGAGAGCAAGCCUAGCAGAGGUACCCAUUCCAUUCCCAGUUUGCUCGGUAGCUGGUGAUUGGAAGACACUCUGCAACAUUA
..(((((((...(((...((((((((((.....)))))).))))...)))...)))))))............. ((((((.......(((((.((((((((........)))))).)).)))))....)))))).......   (-49.00)
```

Reads in GSM314553 (Dicer-knockout mice)

Reads representing snoRNA fragments

```
GACCTCTTGGGATCGCG...........................................................................................................................   1   17   1
GACCTCTTGGGATCGCGTC.........................................................................................................................   1   19   1
GACCTCTTGGGATCGCGTCTGGAG....................................................................................................................   1   24   1
GACCTCTTGGGATCGCGTCTGGA.....................................................................................................................   1   23   1
GACCTCTTGGGATCGCGTCT........................................................................................................................   1   20   1
GACCTCTTGGGATCGC............................................................................................................................   1   16   1
GACCTCTTGGGATCGCGT..........................................................................................................................   3   18   1
....................................................................................................................ATTGGAAGACACTCTGCAACATTA   1   24   1
..........................................................................................................................AGACACTCTGCAACATT.   1   17   2
..........................................................................................................................AGACACTCTGCAACATTA   7   18   1
...........................................................................................................................GACACTCTGCAACATTA   8   17   1
............................................................................................................................ACACTCTGCAACATTA   22  16   4
```

Reads in GSM314557 (Dgcr8-knockout mice)

Reads representing snoRNA fragments

```
GACCTCTTGGGATCGCG...........................................................................................................................   1   17   1
GACCTCTTGGGATCGCGTCTGGAGAGT.................................................................................................................   1   27   1
GACCTCTTGGGATCGCGTCTGGAG....................................................................................................................   1   24   1
GACCTCTTGGGATCGC............................................................................................................................   4   16   1
.....................................................................................................................TTGGAAGACACTCTGCAACAT..   4   21   2
..........................................................................................................................AGACACTCTGCAACATTA   4   18   1
...........................................................................................................................GACACTCTGCAACATTA   11  17   1
............................................................................................................................ACACTCTGCAACATTA   46  16   4
```

Reads representing miRNAs derived from snoRNA

```
......................................................................CTAGCAGAGGTACCCATTCCATTCCC............................................   2   26   1
.......................................................................TAGCAGAGGTACCCATTCCATTCCCA...........................................   1   26   1
.......................................................................TAGCAGAGGTACCCATTCCATTCC.............................................   1   24   1
.......................................................................TAGCAGAGGTACCCATTCCATT...............................................   1   22   1
.......................................................................TAGCAGAGGTACCCATTCCATTCCC............................................   4   25   1
...............................................................................................................TGGTGATTGGAAGACACTCTGCA......   1   23   3
.................................................................................................................GTGATTGGAAGACACTCTGCAAC....   1   23   2
.................................................................................................................GTGATTGGAAGACACTCTGCAA.....   1   22   3
.................................................................................................................GTGATTGGAAGACACTCTGCA......   2   21   3
..................................................................................................................TGATTGGAAGACACTCTGCAA.....   13  21   3
..................................................................................................................TGATTGGAAGACACTCTGCAACA...   32  23   2
..................................................................................................................TGATTGGAAGACACTCTGCAACAT..   3   24   2
..................................................................................................................TGATTGGAAGACACTCTGCA......   3   20   3
..................................................................................................................TGATTGGAAGACACTCTGCAAC....   5   22   2
```
