# Supplementary material for: Noncanonical MicroRNAs and Endogenous siRNAs in Lytic Infection of Murine Gammaherpesvirus
Source: PLoS One. 2012 Oct 26;7(10):e47863. doi: 10.1371/journal.pone.0047863 (PMC3482243; doi:10.1371/journal.pone.0047863)

Supplemental Figure S4. Examples of known and novel snoRNA-derived miRNAs bearing atypical folding structures. (A) Folding structure of snoRNA HBI-100 carrying an annotated miRNA,mmu-miR-1843 with 4.5 unpaired bases in the central bulge, indicated inside the red ellipsis. (B) Folding structure of SNORA1 carrying novel sno-miR-#9 with 5 unpaired bases in the central bulges.

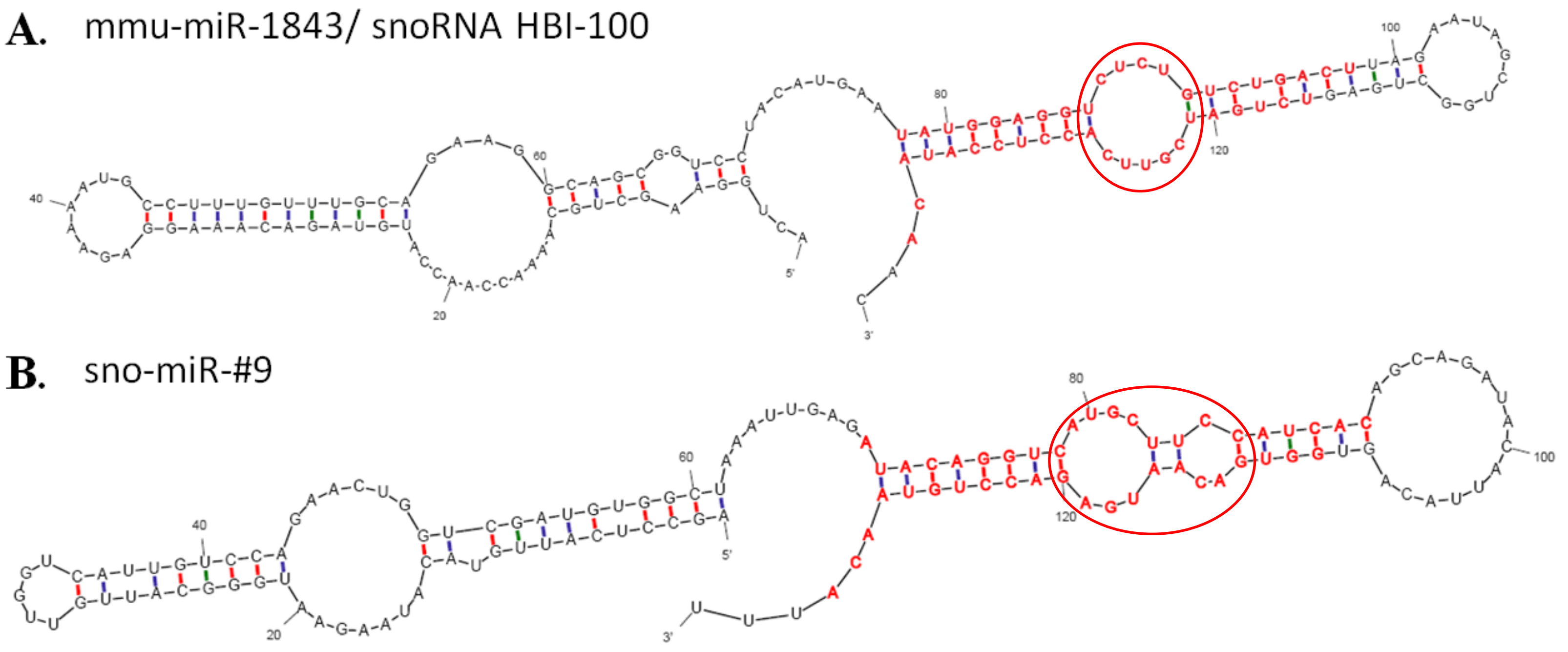

Supplement: Figure S4 — Examples of known and novel snoRNA-derived miRNAs bearing atypical folding structures. (A) Folding structure of snoRNA HBI-100 carrying an annotated miRNA,mmu-miR-1843 with 4.5 unpaired bases in the central bulge, indicated inside the red ellipsis. (B) Folding structure of SNORA1 carrying novel sno-miR-#9 with 5 unpaired bases in the central bulges. (PDF) [file pone.0047863.s004.pdf]
