# Supplementary material for: Noncanonical MicroRNAs and Endogenous siRNAs in Lytic Infection of Murine Gammaherpesvirus
Source: PLoS One. 2012 Oct 26;7(10):e47863. doi: 10.1371/journal.pone.0047863 (PMC3482243; doi:10.1371/journal.pone.0047863)
Supplement: Figure S6 — Sequence reads mapped to the (A) mmu-miR-702 and (B) mmu-miR-5132 hairpins. Sequence in blue represents intron region, while in yellow represents flanking exon regions. Reads comprising GU or AG dinucleotide, which is characteristic of intron splice sites, were marked in red. (PDF) [file pone.0047863.s006.pdf]

Supplemental Figure S6. Sequence reads mapped to the (**A**) mmu-miR-702 and (**B**) mmu-miR-5132 hairpins. Sequence in blue represents intron region, while in yellow represents flanking exon regions. Reads comprising GU or AG dinucleotide, which is characteristic of intron splice sites, were marked in red.

A. mmu-miR-702 region

```
CGGGACAAGGUGAGUGGGGUGGUUGGCAUGGGUUGCCCAUGGGGACUCGACGCUGUGCCCACAGCCUCCUGAUGUCCUCCUCACGCAUGCCCACCCUUUACCCCGCUCCAGGGCAUCUU
.((..(...(.((((((((((((..((..((((((((....((((((((((.(((((....))))).))..)).)))).))....))).)))))))..))))))))))))...)..)).. (-50.30)
```

Reads from GSE36639 (MHV68-infected)

```
.........GUGAGUGGGGUGGUUGGCA........................................................................................... 1 19 1
.........GUGAGUGGGGUGGUUGGCAUG......................................................................................... 9 21 1
..........UGAGUGGGGUGGUUGGCAUGGGU...................................................................................... 1 23 1
.......................................................................................UGCCCACCCUUUACCCCGCUCCAG........ 2 24 1
```

Reads from GSM314557

```
.........GUGAGUGGGGUGGUUGGCAUG......................................................................................... 25 21 1
.........GUGAGUGGGGUGGUUGG............................................................................................. 3 17 1
.........GUGAGUGGGGUGGUUGGCA........................................................................................... 9 19 1
..........UGAGUGGGGUGGUUGGCA........................................................................................... 1 18 1
..........UGAGUGGGGUGGUUGGCAUGGG....................................................................................... 2 22 1
..........UGAGUGGGGUGGUUGGCAU.......................................................................................... 2 19 1
......................................................................................AUGCCCACCCUUUACCCCGCUC........... 1 22 1
.......................................................................................UGCCCACCCUUUACCCCGCUCCA......... 10 23 1
.......................................................................................UGCCCACCCUUUACCCCGC............. 1 19 1
.......................................................................................UGCCCACCCUUUACCCCG.............. 1 18 1
.......................................................................................UGCCCACCCUUUACCCCGCU............ 3 20 1
.......................................................................................UGCCCACCCUUUACCCC............... 4 17 2
.......................................................................................UGCCCACCCUUUACCCCGCUC........... 5 21 1
.......................................................................................UGCCCACCCUUUACCCCGCUCCAG........ 60 24 1
.......................................................................................UGCCCACCCUUUACCCCGCUCC.......... 9 22 1
```

B. mmu-miR-5132 region

```
CCGGCGUGGGGUGGUGGACUCAGGCUUCUGGAGCUGAAGCCUGAUGUUCCCCAUCCCGCAG
.....((((((((.((((((((((((((.......))))))))).)))).)))))))))).. (-38.40)
```

Reads from GSE36639 (MHV68-infected)

```
...GCGTGGGGTGGTGGACTCAGG.....................................  1   21  1
....CGTGGGGTGGTGGACTCAGGCT...................................  1   22  1
....CGTGGGGTGGTGGACTCAGGCTT..................................  1   23  1
....CGTGGGGTGGTGGACTCAG......................................  1   19  1
....CGTGGGGTGGTGGACTCAGGC....................................  4   21  1
.....GTGGGGTGGTGGACTCAGG.....................................  2   19  1
......TGGGGTGGTGGACTCAGGCTTC.................................  19  22  1
......TGGGGTGGTGGACTCAGGC....................................  1   19  1
......TGGGGTGGTGGACTCAGGCT...................................  1   20  1
......TGGGGTGGTGGACTCAGGCTT..................................  4   21  1
```

Reads from GSM314557

```
...GCGTGGGGTGGTGGACTCAGG.....................................  1   21  1
....CGTGGGGTGGTGGACTCAGGC....................................  1   21  1
....CGTGGGGTGGTGGACTCAGGCT...................................  1   22  1
....CGTGGGGTGGTGGACTCAGGCTTCT................................  1   25  1
....CGTGGGGTGGTGGACTCAGGCTT..................................  1   23  1
....CGTGGGGTGGTGGACTCAGG.....................................  1   20  1
....CGTGGGGTGGTGGACTCA.......................................  1   18  1
....CGTGGGGTGGTGGACTC........................................  4   17  1
.....GTGGGGTGGTGGACTCAGG.....................................  1   19  1
......TGGGGTGGTGGACTCAGGCT...................................  1   20  1
......TGGGGTGGTGGACTCAGGCTTC.................................  7   22  1
..........TGGTGGACTCAGGCTTC..................................  1   17  2
.....................................AGCCTGATGTTCCCCATCCCGCA.  1   23  1
.......................................CCTGATGTTCCCCATCCCGCAG  1   22  1
........................................CTGATGTTCCCCATCCCGCA.  1   20  1
```
