# Supplementary material for: Noncanonical MicroRNAs and Endogenous siRNAs in Lytic Infection of Murine Gammaherpesvirus
Source: PLoS One. 2012 Oct 26;7(10):e47863. doi: 10.1371/journal.pone.0047863 (PMC3482243; doi:10.1371/journal.pone.0047863)

Supplemental Figure S7. Six of 17 previously annotated cis-NATs in the previous study[24] appeared in the current MHV68-infected data. All of the loci are based on UCSC mouse reference genome (version mm8). Mapping of reads and numbers of reads (log2 based) in current data set (GSE36639) and in oocyte (GSM261957) are shown in the top two tracks. .RefSeq and Ensembl Gene annotations, repeat elements and conservation scores are shown (see USCS genome browser for details of track information).

1. chr14_48088967_48178294

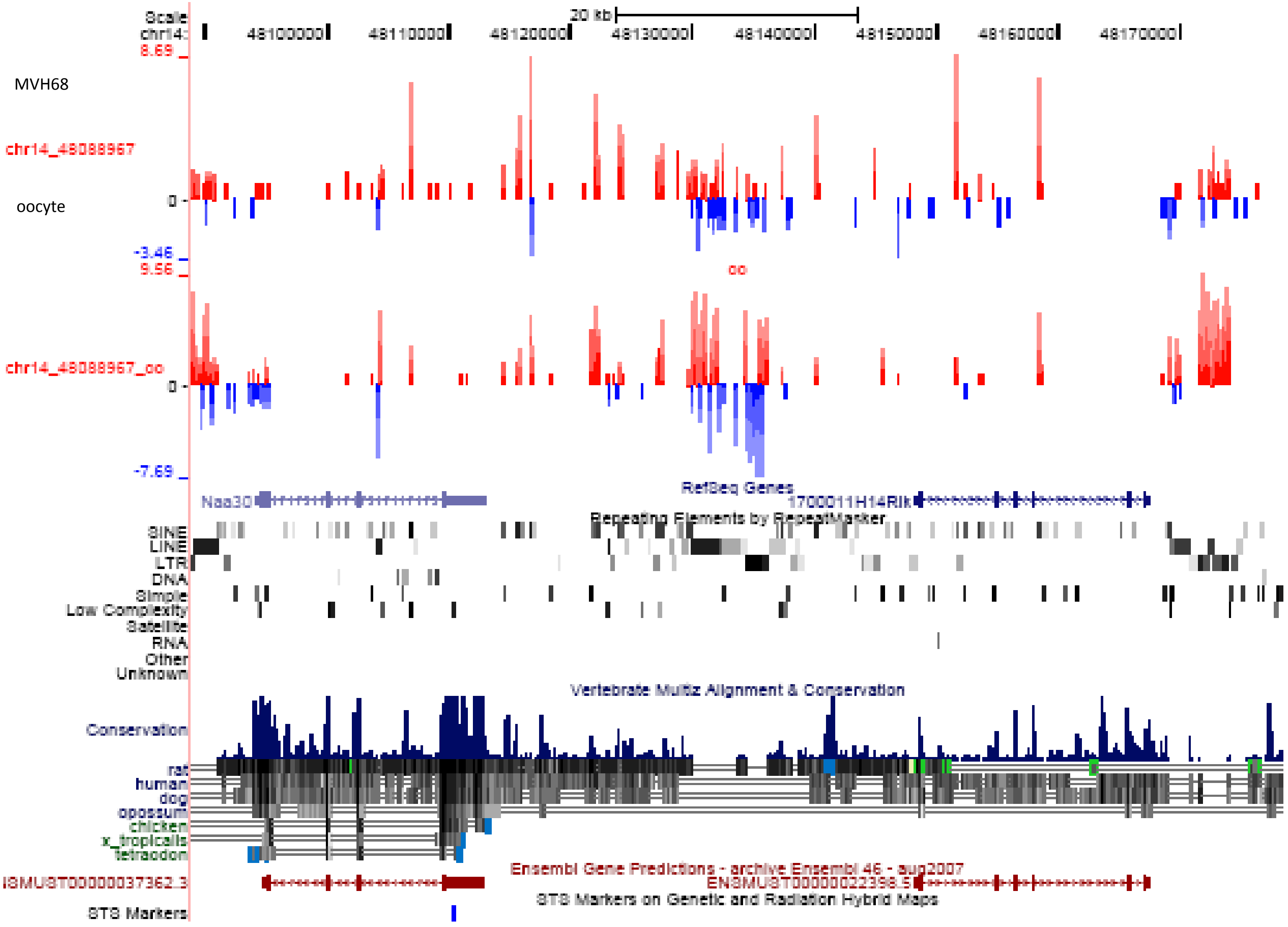

2. chr3_103149689_103158115

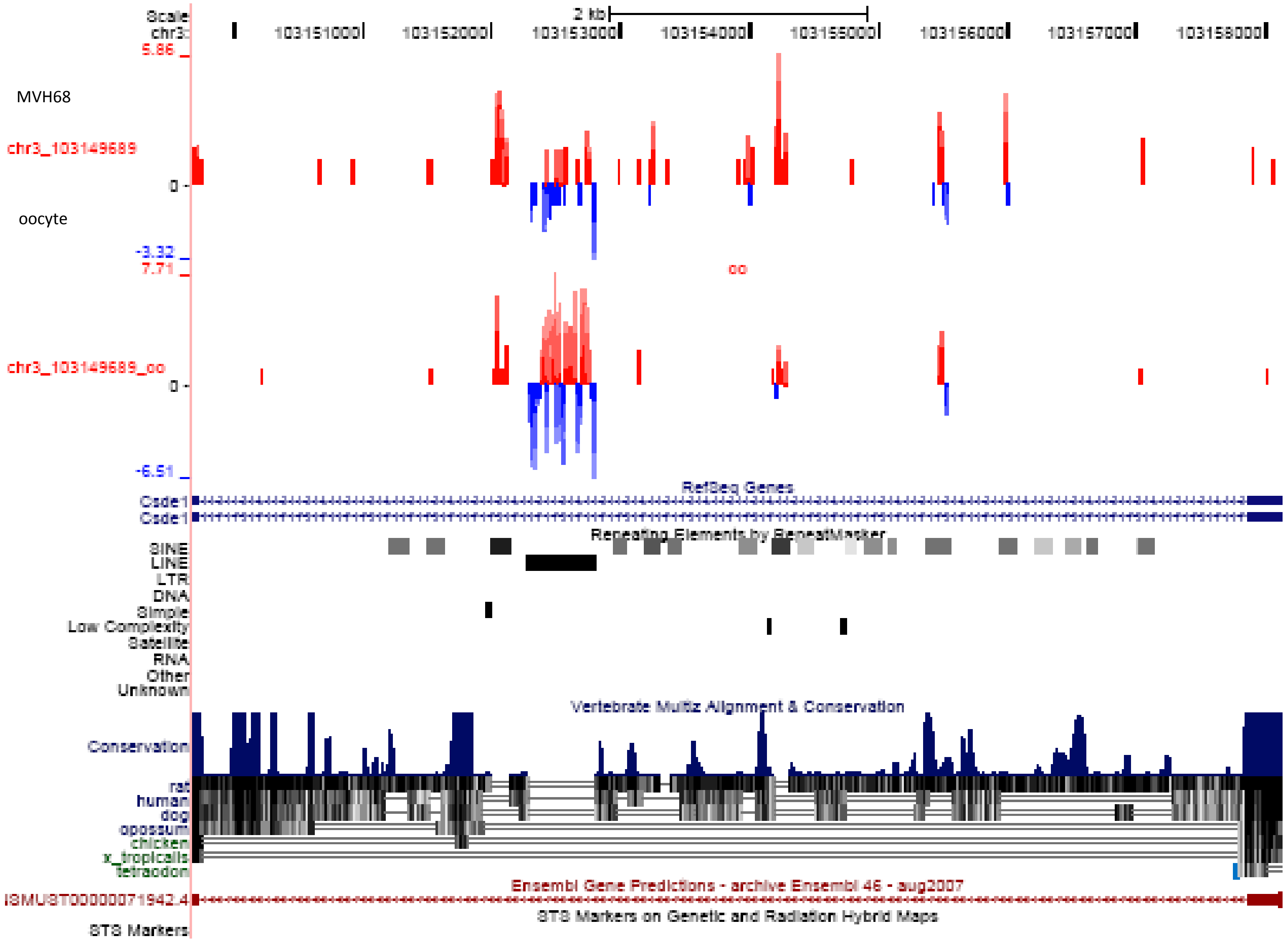

3. chr4:131168448-131195984

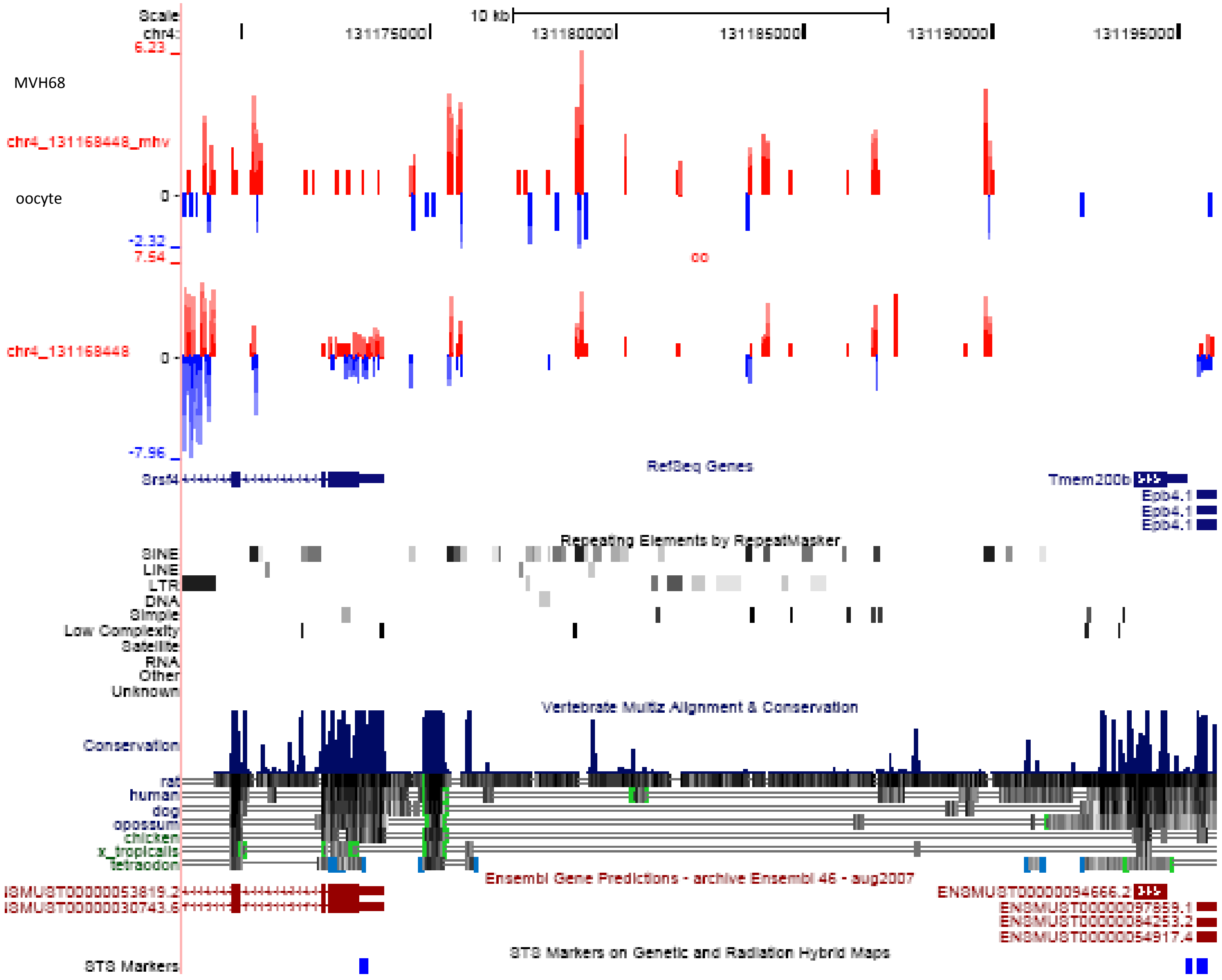

4. chr5_114103753_114204341

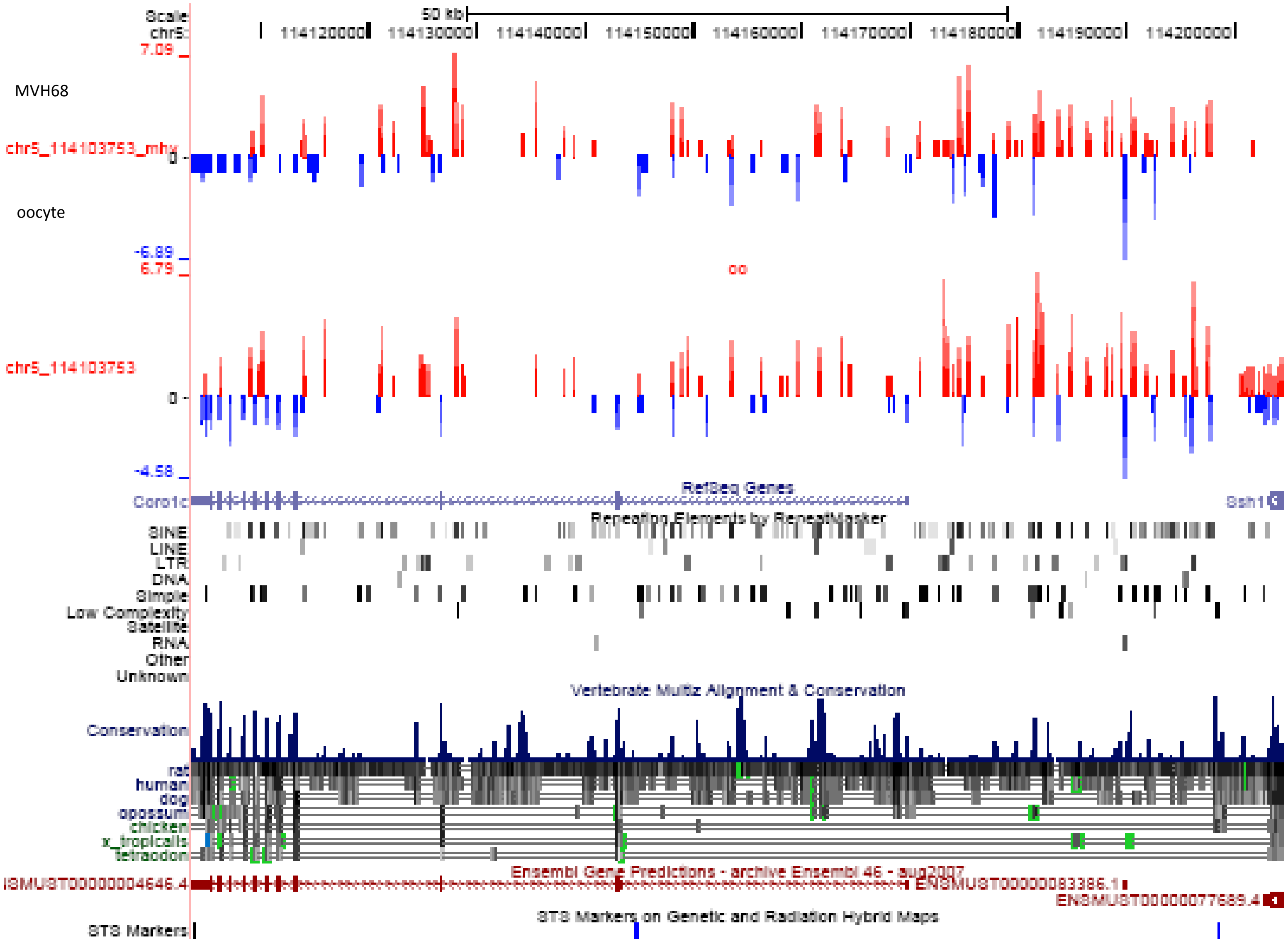

5. chr6_95209508_95276509

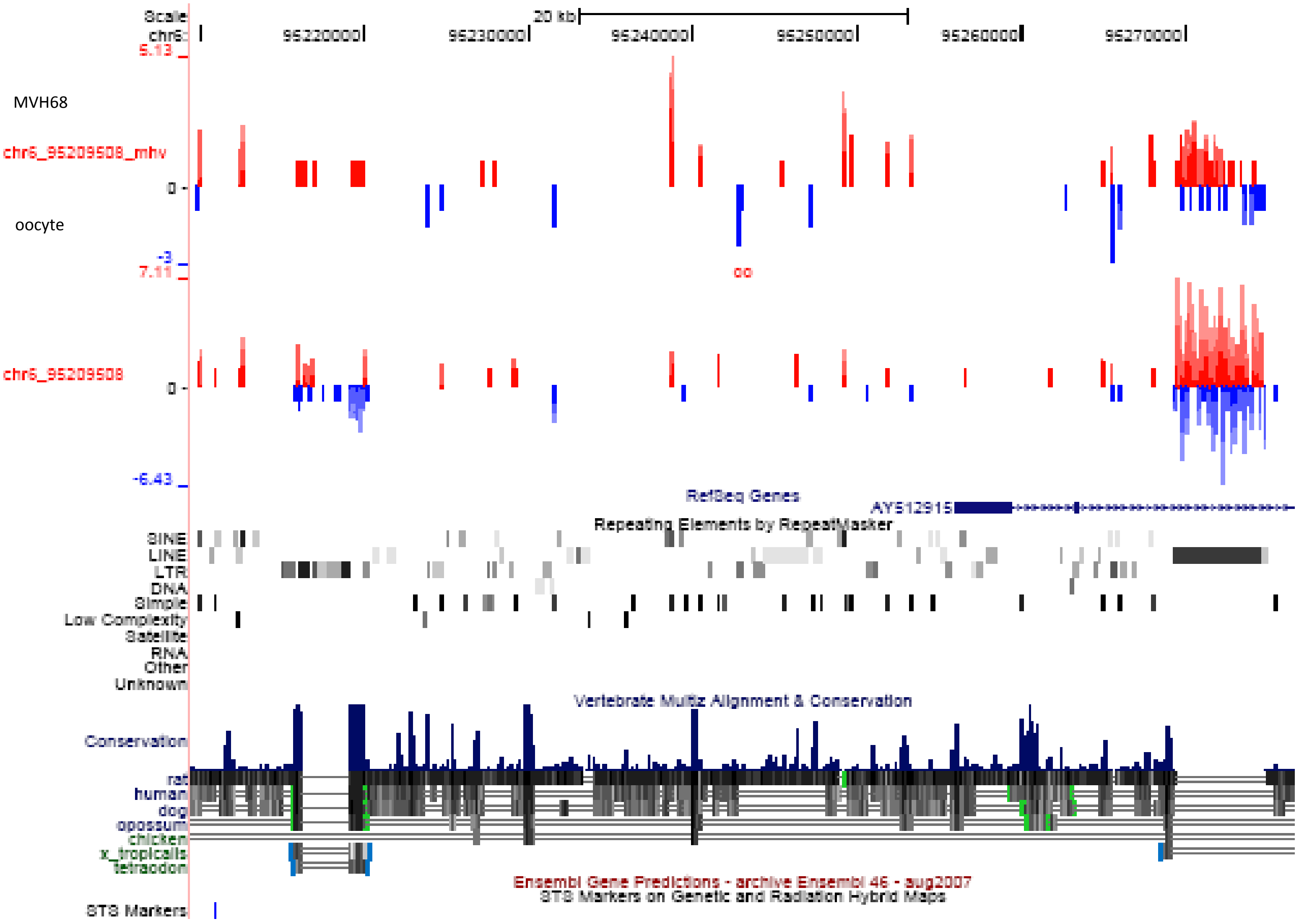

6. chr8_126304234_126307701

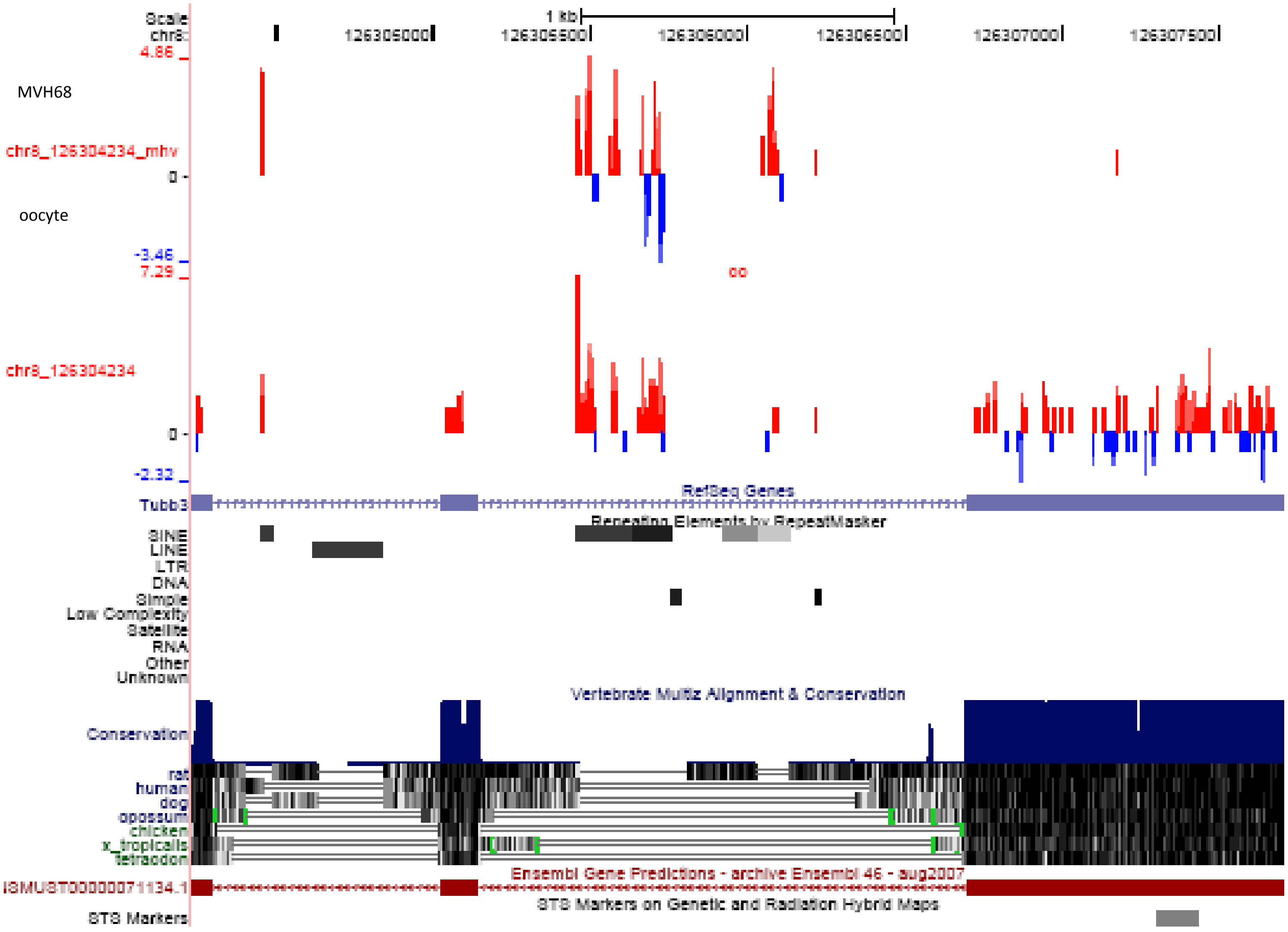

Supplement: Figure S7 — Six of 17 previously annotated cis-NATs in the previous study [24] appeared in the current MHV68-infected data. All of the loci are based on UCSC mouse reference genome (version mm8). Mapping of reads and numbers of reads (log2 based) in current data set (GSE36639) and in oocyte (GSM261957) are shown in the top two tracks. RefSeq and Ensembl Gene annotations, repeat elements and conservation scores are shown (see USCS genome browser for details of track information). (PDF) [file pone.0047863.s007.pdf]
