# Supplementary material for: Noncanonical MicroRNAs and Endogenous siRNAs in Lytic Infection of Murine Gammaherpesvirus
Source: PLoS One. 2012 Oct 26;7(10):e47863. doi: 10.1371/journal.pone.0047863 (PMC3482243; doi:10.1371/journal.pone.0047863)

Supplemental Figure S8. The relationship between the copy number and the percentage of all genomic-aligned reads included

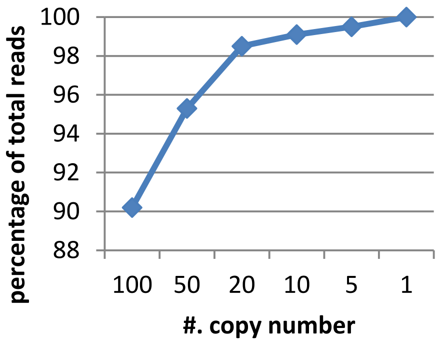

Supplement: Figure S8 — The relationship between the copy number and the percentage of all genomic-aligned reads included. (PDF) [file pone.0047863.s008.pdf]
