# Supplementary material for: Noncanonical MicroRNAs and Endogenous siRNAs in Lytic Infection of Murine Gammaherpesvirus
Source: PLoS One. 2012 Oct 26;7(10):e47863. doi: 10.1371/journal.pone.0047863 (PMC3482243; doi:10.1371/journal.pone.0047863)
Supplement: Figure S5 — A murine miRNA derived from tRNA-SerAGA. (A) Alignment of reads representing the tRNA fragments and miRNA sequences. The tRNA is annotated in blue with the putative Pol III transcription terminal signal (in orange). Untemplated reads derived from tRNA CCA addition end with blue characters, while miRNA reads are annotated in red. The arrows indicate the RNase P (left) and RNase Z (right) processing. (B) The tRNA folding structure (left) and the alternative miRNA hairpin structure (right) with the miRNA annotated in red, with folding energy being presented below each structure. (C) Multiple sequences alignments of tRNA-SerAGA sequence among eight mammalian species with the miRNA indicated in the red rectangle. (PDF) [file pone.0047863.s005.pdf]

Supplemental Figure S5. A murine miRNA derived from tRNA-SerAGA. (**A**) Alignment of reads representing the tRNA fragments and miRNA sequences. The tRNA is annotated in blue with the putative Pol III transcription terminal signal (in orange). Untemplated reads derived from tRNA CCA addition end with blue characters, while miRNA reads are annotated in red. The arrows indicate the RNase P (left) and RNase Z (right) processing. (**B**) The tRNA folding structure (left) and the alternative miRNA hairpin structure (right) with the miRNA annotated in red, with folding energy being presented below each structure. (**C**) Multiple sequences alignments of tRNA-SerAGA sequence among eight mammalian species with the miRNA indicated in the red rectangle.

A.

```
   chr13:23592811-23592912 [-]
5' CACUUGUAGUCGUGGCCGAGUGGUUAAGGCGAUGGACUAGAAAUCCAUUGGGGUCUCCCCGCGCAGGUUCGAAUCCUGCCGACUACGAUUGUGGGUUUUUGU 3'
   ((((((((((((..(((.........))).(((((.......))))).((((...))))..(((((.......))))))))))))))..))).........
      Reads derived from tRNA folding
   ....GUAGUCGUGGCCGAGUGG..........................................RNase Z →.....................   710   18   10
   ....GUAGUCGUGGCCGAGUGGUUAAGG....................................................................   141   24   9
   ....GUAGUCGUGGCCGAGUGGUUAAGGC...................................................................   146   25   9
   ...............................................................GUUCGAAUCCUGCCGACUACGCCA.........   2     24   0
   ....← RNase P .....................................................UCGAAUCCUGCCGACUACGCCA.........   62    22   0
   ........................................................................AUCCUGCCGACUACGCCA.........   175   18   0
   .........................................................................UCCUGCCGACUACGCCA.........   66    17   0
      Reads derived from miRNA folding
   .(((..(((((((((.((.((((((..(((.(((((.......))))).((((...)))).....)))..))))..)))).)))))))))..))).....
   ...................................................................CCUGCCGACUACGAUUGUGG........   1     20   1
   ....................................................................CUGCCGACUACGAUUGUGG........   2     19   1
   .....................................................................UGCCGACUACGAUUGUGGGU.....   3     20   1
   .....................................................................UGCCGACUACGAUUGUGGGUU....   3     21   1
   .....................................................................UGCCGACUACGAUUGUGGG......   3     19   1
                                                                                                    #reads length #loci
```

B.

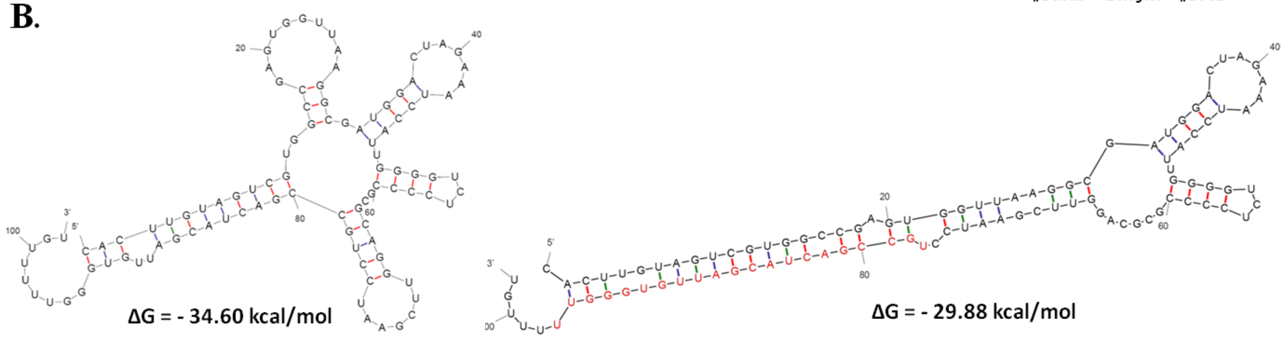

C.

```
mouse      CACTTGTAGTCGTGGCCGAGTGGTTAAGGCGATGGACTAGAAATCCATTGGGGTCTCCCCGCGCAGGTTCGAATCCTGCCGACTACGATTGTGGGTTTTTGT   102
rat        CACTTGTAGTCGTGGCCGAGTGGTTAAGGCGATGGACTAGAAATCCATTGGGGTCTCCCCGCGTAGGTTCGAATCCTGCCGACTACGGCTGTGGGCTTTTGT   102
human      TATCTGTAGTCGTGGCCGAGTGGTTAAGGCGATGGACTAGAAATCCATTGGGGTCTCCCCGCGCAGGTTCGAATCCTGCCGACTACGGCAGTGGGTTTTTGC   102
orangutan  TACCTGTAGTCGTGGCCAAGTGGTTAAGGCGATGGACTAGAAATCCATTGGGGTCTCCCCGCGCAGGTTCGAATCCTGCCGACTACGGTAGTGGATTTTTGC   102
dog        GTCATGTAGTCGTGGCCGAGTGGTTAAGGCGATGGACTTGAAATCCATTGGGGTCTCCCCGCGCAGGTTCGAATCCTGCCGACTACGGAGTAGTGTTTTTCT   102
horse      CATCTGTAGTCGTGGCCGAGTGGTTAAGGCGATGGACTAGAAATCCATTGGGGTCTCCCCGCGCAGGTTCGAATCCTGCCGACTACGGAAGTTGACTTTTAC   102
opossum    GGGGTGTAGTCGTGGCCGAGTGGTTAAGGCGGTGGACTAGAAATCCATTGGGGTCTCCCCGCGCAGGTTCGAATCCTGCCGACTACGAGTGTTGGTTTTTCT   102
cons           ************* ************* ****** ************************ **********************        ****     102
```
